# Quantum-like representation of neuronal networks' activity: modeling “mental entanglement”

**DOI:** 10.3389/fnhum.2025.1685339

**Published:** 2025-12-09

**Authors:** Andrei Khrennikov, Makiko Yamada

**Affiliations:** 1 Center for Mathematical Modeling in Physics and Cognitive Sciences, Linnaeus University, Växjö, Sweden; 2 Institute for Quantum Life Science, National Institutes for Quantum Science and Technology, Chiba, Japan; 3 Institute for Quantum Medical Science, National Institutes for Quantum Science and Technology, Chiba, Japan; 4 Graduate School of Science and Engineering, Chiba University, Chiba, Japan

**Keywords:** quantum-like modeling, neuronal networks, mental entanglement, decision making, EEG/MEG technique

## Abstract

*Quantum-like modeling* (QLM)—quantum theory applications outside of physics—are intensively developed with applications in biology, cognition, psychology, and decision-making. For cognition, QLM should be distinguished from quantum reductionist models in the spirit of Hameroff and Penrose, as well as Umezawa and Vitiello. QLM is not only concerned with just quantum physical processes in the brain but also with QL information processing by macroscopic neuronal structures. Although QLM of cognition and decision-making has seen some success, it suffers from a knowledge gap that exists between oscillatory neuronal network functioning in the brain and QL behavioral patterns. Recently, steps toward closing this gap have been taken using the generalized probability theory and prequantum classical statistical field theory (PCSFT)—a random field model beyond the complex Hilbert space formalism. PCSFT is used to move from the classical “*oscillatory cognition*” of the neuronal networks to QLM for decision-making. In this study, we addressed the most difficult problem within this construction: QLM for entanglement generation by classical networks, that is, “*mental entanglement*.” We started with the observational approach to entanglement based on operator algebras describing “local observables” and bringing into being the tensor product structure in the space of QL states. Moreover, we applied the standard states entanglement approach: entanglement generation by spatially separated networks in the brain. Finally, we discussed possible future experiments on “mental entanglement” detection using the EEG/MEG technique.

## 1 Introduction

Intensive development of quantum information theory has transformed the perspective of quantum studies toward an information-based approach to physics. In particular, numerous information–theoretic interpretations of quantum mechanics have been proposed (Khrennikov, 2024b). These interpretations exert both foundational and technological influence. This informatization of quantum physics has also stimulated applications of the methodology and formalism of quantum theory beyond physics, extending to biology, cognition, psychology, decision-making, economics, finance, and the social and political sciences [see, e.g., monographs (Khrennikov, 2004, 2010; Busemeyer and Bruza, 2024; Plotnitsky and Haven, 2023; Asano et al., 2015; Bagarello, 2019; Khrennikov, 2023b) and reviews (Pothos and Busemeyer, 2022; Khrennikov, 2023a)]—a direction commonly termed *quantum-like modeling* (QLM). In this article, we focus on QLM in the domains of cognition and decision-making.

Here, QLM must be clearly distinguished from quantum reductionist models advanced by Hameroff (1994), Penrose (1989), Vitiello (2001), Vallortigara and Vitiello (2024), Igamberdiev (2004), Igamberdiev and Brenner (2021), who associated cognition and consciousness with quantum physical processes in the brain. Hameroff and Penrose emphasized microtubules, Vitiello referred to quantum field theory and long-range correlations in the brain, and Igamberdiev linked cognition to quantum processes in cells. In contrast, QLM of cognition does not concern quantum physical processes in the brain but rather quantum-like information processing by macroscopic neuronal networks.

QLM of cognition and decision-making has been successfully developed; it mathematically describes non-classical features of cognitive phenomena, such as “interference and entanglement of minds.” It resolves numerous paradoxes of decision theory and models basic cognitive effects, including conjunction, disjunction, order, response replicability, and Zeno effects [see the mentioned works and articles (Atmanspacher et al., 2004; Wang and Busemeyer, 2013; Wang et al., 2014; Khrennikova, 2014; Ozawa and Khrennikov, 2020, 2021; Tsuchiya et al., 2025)]. QLM has highlighted the contextuality of cognitive phenomena by applying advanced quantum contextuality theory and, in particular, the machinery of Bell inequalities (Conte et al., 2008; Cervantes and Dzhafarov, 2018; Basieva et al., 2019; Gallus et al., 2022, 2023; Khrennikova, 2025). QLM has introduced a novel perspective on rationality vs. irrationality and violations of the Savage Sure Thing Principle (Busemeyer and Bruza, 2024), framed within probabilistic and logical approaches, such as violations of Bayesian updating (Ozawa and Khrennikov, 2020, 2021), the formula of total probability (Khrennikov, 2004, 2006b, 2010; Asano et al., 2015; Khrennikova, 2013), and classical Boolean logic, while incorporating quantum logic (Ozawa and Khrennikov, 2023; Gunji et al., 2020; Gunji and Nakamura, 2022). QLM has successfully described statistical data on bistable perception of ambiguous figures (Atmanspacher et al., 2004; Atmanspacher and Filk, 2013; Conte et al., 2009; Khrennikov, 2015, 2004), which has been applied to biological evolution (including genetic and epigenetic mechanisms; Asano et al., 2013, 2015; Khrennikov et al., 2024), and, more recently, to aesthetic experiences during book reading (Fuyama, 2023, 2024).

Nevertheless, cognitive QLM faces a gap between theoretical descriptions of classical oscillatory neuronal networks in the brain (e.g., the phase space description of harmonic oscillators) and the quantum-like representation of mental states and observables (e.g., density and Hermitian operators). Thus, it remains a phenomenological framework.

Recently, progress toward bridging this gap has been achieved (Khrennikov et al., 2025a) within the framework of *prequantum classical statistical field theory* (PCSFT)—a random field model generating the complex Hilbert space formalism for states and observables (Khrennikov, 2005, 2006a, 2014a, 2024a). PCSFT has been employed to connect classical “oscillatory cognition” of neuronal networks with QLM for decision-making.^1^

In this article, we proceed to the most difficult problem within such construction: creation of QLM for the *generation of entanglement by classical networks*—the problem of “*mental entanglement*.”

At present, our approach to “mental entanglement” is purely theoretical and is based on the mathematical formalism of quantum theory. Research in brain science has, of course, demonstrated strong correlations between neuronal activities in different regions of the brain, and these findings provide indirect support for our theoretical model. A natural question arises: can “mental entanglement” be verified experimentally? Is it possible to propose experimental tests grounded in the formal mathematical notions of observational (Sections 6 and 7) and state (Section 8) entanglement? This complex issue is discussed in Sections 10 and 11, where we suggest EEG-based tests and explore parallels between quantum and electrophysiological measures of entanglement. We believe that these considerations may help to establish connections with researchers in neurophysiology and potentially encourage collaboration in designing new experiments on “mental entanglement.”

We now outline the content of this article. In Section 2, we discuss the basics of QLM, the mathematical formalism of QL decision making, and, as an example, application to the order effect. This effect is easily structured within the QL scheme; this was one of the first successful realizations of this scheme. In Section 2, we formulate the fundamental conjecture on QL information processing in the brain. This article aims to justify (at least partially) this conjecture. Section 3 presents the basic construction for transitioning from oscillatory dynamics of neuronal networks in the brain to the quantum-like (QL) representation. Section 4 links classical and quantum realizations of observables on neuronal networks. Section 5 concerns the mathematical model for connecting the dynamics of a system of coupled harmonic oscillations with QL state dynamics described by the Schrödinger equation. Section 6 addresses a specific approach to the notion of entanglement, treating it as the entanglement of observables. In Section 7, this approach is applied to the entanglement of observables on neuronal circuits. In Section 8, we examine the standard approach to entanglement as state entanglement and its application to modeling mental entanglement. In Section 9, we discuss the role of ephaptic coupling in the generation of correlations between neuronal circuits in the brain. Section 10 concerns possible experimental verification of mental entanglement. This section deserves the special attention of experts in experimental cognitive science and neuroscience. Here, we discuss concrete experimental tests of mental entanglement based on classical electroencephalogram (EEG)/magnetoencephalography (MEG) measurement techniques, including comparative analysis with classical EEG-based approaches to functional connectivity in neuroscience; we also analyze the possibility of creating and detecting entanglement in *in vitro* neuronal networks. Section 11 describes the main quantitative measures of entanglement that can be used in experimental tests. Section 12 is devoted to foundational questions of entanglement theory, where we compare quantum, classical, and mental entanglement, coupling to “superstrong quantum correlations” and the Bell Inequality. Conceptual differences between classical and QL frameworks are discussed in Section 13. Section 14 provides a general discussion on the proposed model. Section 5 concerns the mathematical model for connecting the dynamics of a system of coupled harmonic oscillations with QL state dynamics described by the Schrödinger equation.

In physics, entanglement is one of the most intriguing and complex quantum phenomena. Typically, entanglement is treated as *state entanglement*. In the mathematical formalism of quantum theory, a pure state |ψ〉 of a compound system *S* = (*S*_1_, *S*_2_) is given by a normalized vector belonging to the tensor product of two complex Hilbert spaces, H = H_1_ ⊗ H_2_. A state |ψ〉 ∈ H is called entangled if it cannot be factorized, that is, it does not have the form |ψ〉 = |ψ〉_1_ ⊗ |ψ〉_2_, where |ψ〉_*i*_ ∈ H_*i*_, *i* = 1, 2.

Another less familiar approach to the notion of entanglement is *observation entanglement* (Zanardi, 2001); (Zanardi et al., 2004); (Basieva and Khrennikov, 2022); (Khrennikov and Basieva, 2023), based on considering “local algebras” of cross-commuting observables **A**_1_ and **A**_2_. In this view, a tensor product structure on the state space is not preassigned but is generated by these operator algebras. The same state space H can be endowed with multiple tensor–product structures corresponding to different choices of operator algebras. In this article, we employ both approaches to the notion of entanglement.

Since observation entanglement is less familiar and not widely applied, we complete the article with a special section devoted to this notion, Section 6. We then proceed to the entanglement of observables on neuronal networks in Section 7.

State entanglement generated by a compound neuronal network *S* = (*S*_1_, *S*_2_) is discussed in Section 8. Such entanglement and its generation by interacting neuronal networks are of neurophysiological interest. To identify its roots, we must look deeper into brain architecture and communication between neuronal circuits, including those not physically connected through axonal–dendritic pathways. Here, we emphasize the role of electromagnetic signaling, particularly ephaptic coupling between neuronal structures in the brain (Section 9). However, discussion of state entanglement in neuronal networks (Section 9) is primarily theoretical, as experimental detection remains far from reach. Observation entanglement (Sections 6 and 7) is more promising for experimentation. The central challenge is identifying observables on neuronal networks capable of exhibiting such entanglement.

This *article is conceptual* and aims to demonstrate the possibility of modeling generally non-local correlations in the brain that are mathematically described as quantum state entanglement. At present, the biophysical mechanism of generating such states is not completely clear. This is the place to emphasize that “mental non-locality,” mathematically described as entanglement, is unrelated to the so-called spooky action at a distance often associated with “quantum non-locality.” Unlike quantum physics experiments on spatially separated systems, such as two photons 100 km apart, in cognitive science, we study a small physical system, the brain. Electromagnetic signals connect any two points in the brain almost immediately. Thus, *biological non-locality expressed via entanglement is classical non-locality generated by electromagnetic signaling between neuronal circuits*. Mental entanglement is the mathematical description of non-local correlations between observations performed on activated neuronal circuits.

We note that in article (Khrennikov et al., 2025a), entanglement of neuronal networks was only mentioned in an unconventional framework that avoided tensor products, instead employing the classical Descartes product (see Khrennikov, 2005, 2006a, 2014a, 2024a). This approach may be interesting from the perspective of classical oscillatory cognition. However, by using it, we lose connection with the notion of entanglement as defined in quantum information theory.

We also speculate that by exploring the QL representation of mental states and observables, the brain may realize so-called *quantum-inspired algorithms* (Huynh et al., 2023) and thereby achieve essential enhancement of computational power (Effenberger et al., 2025). In such algorithms, the ability to generate entangled states is important.

We remark that QLM based on the QL representation of EEG signals is already applied in medical diagnostics of neurological disorders, including depression, epilepsy, and schizophrenia (Shor et al., 2021b, 2023). Such diagnostics work unexpectedly well, but in the absence of a theoretical justification. Mental entanglement can serve as the theoretical basis for EEG-based QL diagnostics, as it mathematically describes non-local information processing in the brain. The observed violation of the Clauser–Horne–Shimony–Holt (CHSH) inequality for EEG signals (transformed into dendrograms with clustering algorithms) can also be connected to mental entanglement.

We also highlight several alternative approaches to modeling neuronal state dynamics (Eberly et al., 2016; Bardella et al., 2024a; Buice, 2009; Gentili, 2021; Bardella et al., 2024b; Buice, 2007; Dick and Helias, 2024). To begin with, article (Buice, 2009) explicitly connects quantum probability with fuzzy logic and Bayesian inference—an approach that aligns closely with the conceptual foundations of Quantum-Like Modeling (QLM). In addition, a series of studies (Eberly et al., 2016; Bardella et al., 2024a; Gentili, 2021; Bardella et al., 2024b; Buice, 2007; Dick and Helias, 2024) developed field-theoretic models of neural activity, formulated both in classical and quantum frameworks, often on lattice structures. Some of these works employ tools from operator theory—for instance, article (Bardella et al., 2024a) utilizes the formalism of creation and annihilation operators to solve a reaction–diffusion–type master equation. In article (Dick and Helias, 2024), a Euclidean field model of neural activity is introduced, and the authors discuss the conditions under which a transition to quantum field theory can be achieved via the imaginary-time transformation.

We note that the works (Eberly et al., 2016; Bardella et al., 2024a; Buice, 2009; Gentili, 2021; Bardella et al., 2024b; Buice, 2007) are primarily focused on developing field models—both classical and quantum—of neural activity in the brain. These studies, however, do not aim to provide a neuronal justification for quantum-like cognition and decision making (QCDM; Khrennikov, 2010; Busemeyer and Bruza, 2024; Plotnitsky and Haven, 2023; Asano et al., 2015; Bagarello, 2019; Khrennikov, 2023b; Pothos and Busemeyer, 2022), nor do they attempt to connect theoretical and experimental research in cognitive psychology and decision-making (e.g., the resolution of well-known paradoxes such as those of Allais, Ellsberg, and Machina) with the functioning of neuronal networks in the brain.

In contrast, the present article is devoted to constructing a bridge between brain activity and QCDM. We emphasize that QCDM exhibits genuinely quantum features—such as interference, contextuality, order effects, and response replicability—which can only be adequately described within a quantum formalism, including their various combinations. The applications of QCDM extend far beyond brain research; its methods are widely employed in behavioral economics and finance, as well as in the social and political sciences (Plotnitsky and Haven, 2023).

The article presents an abstract framework grounded in quantum formalism, which may be challenging to read for researchers in neurocognition, particularly experimentalists. To facilitate comprehension, such readers may focus on the introductory part of Section 2 and then proceed directly to Sections 10–14, especially Section 10, which outlines possible experimental studies based on our model of mental entanglement.

## 2 Quantum-like modeling (QLM): Cognition and decision making

Over the past three decades, a growing body of research has revealed that the mathematical and logical instruments traditionally used to describe natural and social phenomena—namely, classical probability theory and Boolean logic are inadequate for capturing the behavior of many complex systems. Empirical evidence across diverse domains, including genetics, epigenetics, evolutionary biology, cognitive science, social, political, economic and financial systems, and game theory, demonstrates systematic deviations from the assumptions of classical rationality and additivity of probability. Contextual dependencies in human reasoning, interference effects in decision-making, and “superstrong” QL correlations expressed in the violation of Bell inequalities, all point to the limits of the conventional analytical paradigm based on classical probability and logic.

These observations have motivated the emergence of an integrative research program commonly known as *Quantum-Like Modeling (QLM)*. This interdisciplinary framework draws upon the formal architecture of quantum theory—its Hilbert space representation, principles of superposition and interference, and the non-commutativity of observables—to model non-classical forms of uncertainty, contextuality, and interdependence in macroscopic information processing. The approach does not posit that cognition or biology in general are *quantum physical* in nature; rather, it employs quantum mathematics and methodology as a flexible representational and inferential tool. Influential contributions to this field include works (Khrennikov, 2024b, 2004, 2010; Busemeyer and Bruza, 2024; Plotnitsky and Haven, 2023; Asano et al., 2015; Bagarello, 2019; Khrennikov, 2023b; Pothos and Busemeyer, 2022; Khrennikov, 2023a; Atmanspacher et al., 2004; Wang and Busemeyer, 2013; Wang et al., 2014; Khrennikova, 2014; Ozawa and Khrennikov, 2020, 2021; Tsuchiya et al., 2025; Conte et al., 2008; Cervantes and Dzhafarov, 2018; Basieva et al., 2019; Gallus et al., 2022, 2023; Khrennikova, 2025; Khrennikov, 2006b; Khrennikova, 2013; Ozawa and Khrennikov, 2023; Gunji et al., 2020; Gunji and Nakamura, 2022; Atmanspacher and Filk, 2013; Conte et al., 2009; Khrennikov, 2015; Melkikh and Khrennikov, 2017; Iriki and Tanaka, 2024; Asano et al., 2013; Khrennikov et al., 2024; Fuyama, 2023, 2024; see also extended bibliography in Pothos and Busemeyer, 2022; Khrennikov, 2023a).

The intellectual foundation of QLM is deeply intertwined with the quantum information revolution, which has extended the interpretive and methodological scope of quantum theory well beyond the boundaries of physics. Unlike traditional quantum mechanics, which focuses on the physical properties of information carriers—such as atoms, electrons, or photons—quantum information theory concerns itself primarily with the *structural principles* governing information processing in such systems. This conceptual shift—from the physics of quantized matter and fields to the theory of information and computation—has a long intellectual lineage, tracing back to John Archibald Wheeler, who introduced and developed the notion of “*it from bit*,” proposing that every physical quantity, every “it,” derives its ultimate significance from bits, binary yes-or-no indications.

Nevertheless, in quantum information theory, the distinctive features of information processing are still understood as emerging from quantum physical substrates. The founders of QLM took a decisive step beyond this constraint by disentangling quantum probability and information theory from their physical foundations, thereby inaugurating a new domain of interdisciplinary inquiry. Within this broader context, one may indeed speak of a *quantum-like revolution* (Plotnitsky and Haven, 2023): a paradigmatic transformation in which the conceptual resources of quantum mechanics have been reconceived as versatile tools for modeling information, cognition, and interaction across complex biological, cognitive, and social systems. This shift has also fostered a growing openness within the quantum community toward non-physical applications of its mathematical and probabilistic formalism.

QLM has proven particularly effective in the domains of cognitive modeling and decision theory—an area often referred to as Quantum-like Cognition and Decision Making (QCDM). Within this framework, QLM provides coherent resolutions to fundamental paradoxes that challenge classical probability-based decision theories, such as the Ellsberg and Machina paradoxes. Moreover, it offers elegant mathematical descriptions of key empirical effects identified in cognitive psychology, including the conjunction, disjunction, order, and response replicability effects, as well as their various combinations. The latter case is especially noteworthy, since modeling such compound effects lies beyond the representational capacity of standard quantum mechanics and instead requires the more sophisticated formal apparatus of quantum information theory.

Now we come to our basic conjecture (Khrennikov, 2004):

**Conjecture**. *At high levels of cognition, the brain operates using a quantum-like (QL) representation, employing QL matrix calculus for information processing*.

QL representation is a meta-representation, a higher-order mental representation of neuronal representation. By this conjecture, the brain represents outputs of neuronal activity as QL states (Section 3) and decisions as observables (Section 4) on such states. At the QL meta-level, the brain dynamically operates not directly with patterns of neural activity but with corresponding QL states.

**Figure 1:**
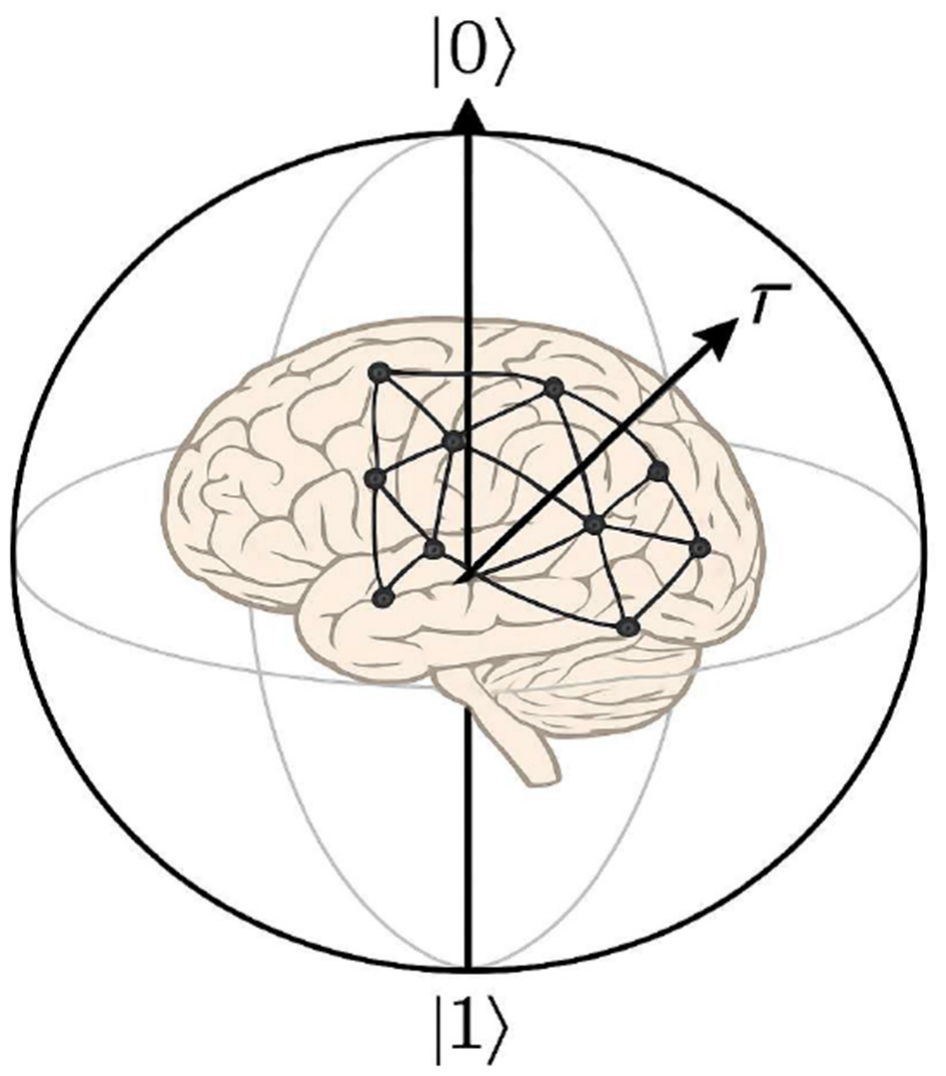
Neuronal networks in the brain generate quantum-like representations of mental states, which can be schematically depicted as vectors on the Bloch sphere, a standard tool for graphically representing qubit states.

This conjecture is strongly supported by the successes of QCDM. However, as noted in the introduction, QCDM remains a phenomenological theory and lacks a well-justified connection to neural processing in the brain. We emphasize once again that our goal is to provide a neuronal-level justification of QCDM, without delving into the microlevel quantum physical processes considered in theories of quantum consciousness. In this article, we take a step toward modeling the neuronal basis of *complex* Hilbert space representations. The coupling between neuronal and QL representations is schematically illustrated in Figure 1. The Bloch sphere visualizes just the simplest quantum states—qubits (and the Bloch ball—mixed qubit states); visualization of higher-dimensional quantum states is difficult. So, in fact the brain should be drawn inside a sphere of a multidimensional complex Hilbert space or maybe even infinite infinite-dimensional space (by taking into account the brain's operation with electromagnetic fields).

### 2.1 Quantum formalism for decision making

In this section, we briefly describe the QL framework for decision making (Khrennikov, 2010; Busemeyer and Bruza, 2024). For simplicity, we restrict ourselves to the finite-dimensional case, as is commonly done in most studies of QLM.

Let H be a complex Hilbert space equipped with an inner product 〈·|·〉, and corresponding norm ||ψ||^2^ = 〈ψ|ψ〉. The set of *density operators* on H is denoted by D = D(H). These are positive, Hermitian, trace-one operators. In standard terminology, a density operator represents a *mixed quantum state*, that is, a statistical mixture of pure states.

In the QL framework, the mental or cognitive state of a decision maker is represented by either a normalized vector |ψ〉 ∈ H (a pure state) or, more generally, by a density operator ρ ∈ D(H) (a mixed state). Hence, D serves as the state space of the QL model.

Cognitive observables such as questions, traits, or decision tasks are represented by Hermitian linear operators *A* : H→H. The *spectrum* of *A* corresponds to the possible outcomes of the observation (the possible answers or responses). For a finite-dimensional space H, the spectrum of *A* is discrete, consisting of eigenvalues *x*_1_, *x*_2_*, …,x*_*n*_, which label the possible outcomes.

In a given orthonormal basis of H, the operators can be represented by matrices, and their action on cognitive states can be calculated using standard linear algebra.

**Born's Rule for Expectation Values**. The expected value of an observable *A* in a state represented by a density operator ρ is given by the *Born rule*:


$$\begin{array}{l} <A{>}_{\rho }=Tr\left(\rho A\right). \end{array}$$

This expression is linear in both the state ρ and the observable *A*, similar to how expectations are computed in classical Kolmogorov probability theory, where averages are obtained as integrals with respect to probability measures.

**Spectral Decomposition of Observables**. Any Hermitian operator *A* can be expressed as a weighted sum over its eigenvalues:


$$\begin{array}{l} A=\underset{\alpha }{\sum }\alpha E_{A}\left(\alpha \right), \end{array}$$

where *E*_*A*_(α) is the projection operator onto the eigenspace H_*A*_(α) corresponding to the eigenvalue α.

**Born's Rule for Probabilities (Pure States)**. For a pure state |ψ> ∈ H, the probability of obtaining the measurement outcome *A* = α is given by:


$$\begin{array}{l} P_{\psi }\left(A=\alpha \right)=∥E_{A}\left(\alpha \right)\psi {∥}^{2}=\langle \psi |E_{A}\left(\alpha \right)|\psi \rangle .\; \end{array}$$

**State Update After Measurement (Projection Postulate)**. A measurement with outcome *A* = α induces a *state update* (or back-action) on the system:


$$\begin{array}{l} \rho -\to \rho A=\alpha =\;EA\left(\alpha \right)\rho EA\left(\alpha \right)/Tr\left[E_{A}\left(\alpha \right)\rho \right] \end{array}$$

This completes the brief outline of the QL formalism used for modeling decision making.

### 2.2 Mathematical description of the order effect

One of the central empirical motivations for applying quantum formalism to cognition is the phenomenon known as the *order effect*: the dependence of answers or decisions on the sequence in which questions are asked (see Wang and Busemeyer, 2013; Wang et al., 2014).

In classical probability theory, if two random variables *A* and *B* are jointly defined, the joint probability satisfies *P*(*A* = α*,B* = β) = *P*(*B* = β*,A* = α). However, in the QL framework, when the corresponding cognitive observables do not commute, the order of measurement affects the result.

**Conditional Probabilities in the QL Framework**. Let *A* and *B* be two observables represented by projection-valued measures {*E*_*A*_(α)} and {*E*_*B*_(β)} acting on the same state space. Given an initial cognitive state ρ, the probability of obtaining *A* = α is

P(A = α) = Tr[ρE_*A*_(α)].

After this measurement, the updated state becomes ρ_*A* = α_ as in Equation 4. The conditional probability of then observing *B* = β is defined by:


$$\begin{array}{l} P\left(B=\beta |A=\alpha \right)=Tr\left[\rho_{A=\alpha }E_{B}\left(\beta \right)\right]=Tr\left[E_{B}\left(\beta \right)\rho_{A=\alpha }\right]. \end{array}$$

**Joint Probabilities for Sequential Measurements**. Using Equation 6 and the Born rule, the *joint sequential probability* for first measuring *A* = α and then *B* = β can be written compactly as:


$$\begin{array}{r} P\left(A=\alpha ,B=\beta \right)=P\left(A=\alpha \right)P\left(B=\beta |A=\alpha \right)\\ =Tr\left[E_{B}\left(\beta \right)E_{A}\left(\alpha \right)\rho E_{A}\left(\alpha \right)\right]. \end{array}$$

If the order is reversed (first *B*, then *A*), we obtain the following joint sequential probability:


$$\begin{array}{l} P\left(B=\beta ,A=\alpha \right)=Tr\left[E_{A}\left(\alpha \right)E_{B}\left(\beta \right)\rho E_{B}\left(\beta \right)\right].\; \end{array}$$

**Non-commutativity and the Order Effect**. The difference between these probabilities arises when


$$\begin{array}{l} \left[E_{A}\left(\alpha \right),E_{B}\left(\beta \right)\right]\neq 0.\; \end{array}$$

In that case,


$$\begin{array}{l} P\left(A=\alpha ,B=\beta \right)\neq P\left(B=\beta ,A=\alpha \right).\; \end{array}$$

**Interpretation**. The order effect thus has a simple QL interpretation: *each question or cognitive task acts as a measurement that transforms the underlying mental state*. When the observables do not commute, the order of questioning modifies the interference structure of probabilities and leads to empirically observable asymmetries in responses.

Empirically, such effects have been demonstrated in opinion polling, questionnaire design, and preference reversal situations in which the sequence of cognitive acts changes the statistical outcome (see Wang and Busemeyer, 2013; Wang et al., 2014, and references herein).

## 3 Quantum-like states of neuronal networks

Let *S* be a neuronal network with oscillatory node-circuits numbered as 𝔰_*j*_, *j* = 1, …, *N*. Following (Effenberger et al., 2025), we do not identify nodes of *S* with individual neurons; instead, these are *neuronal circuits* generating oscillations. The state of each circuit 𝔰_*j*_ is mathematically described by the complex variable *z*_*j*_. Why is it complex? To describe oscillatory dynamics, it is convenient to use two (real) variables (*q, p*), where *q* is a coordinate (possibly generalized) and *p* is the momentum—the conjugate variable to *q*. By setting $z=\left(q+ip\right)/\sqrt{2}$, we move from the real phase space to a complex representation. See Section 5 and article (Khrennikov et al., 2025a) for details.

Oscillations in circuits are random—random oscillations (ROs). For each node-circuit 𝔰_*j*_, ROs are expressed as ℂ-valued random variable *z*_*j*_ = *z*_*j*_(ω), where ω is a random parameter. These random variables are correlated. So, *S* generates a random vector *z* = *z*(ω) ∈ ℂ^*N*^. The complex linear space ℂ^*N*^ is endowed with the scalar product


$$\begin{array}{l} \langle v|w\rangle =\sum_{j}^{N}v_{j}w_{j}. \end{array}$$ (1)

This is a complex Hilbert space denoted by the symbol H. Such spaces are basic in quantum theory. So, a complex Hilbert space is naturally coupled to the phase space dynamics (Khrennikov, 2014a; Khrennikov et al., 2025a; see Section 5).

Geometrically, a neuronal network *S* can be represented by a graph *G*_*S*_ with nodes given by neuronal circuits 𝔰_*j*_, *j* = 1, …, *N*. These nodes are connected by edges. In the simplest case, nodes are individual neurons and edges are axon–dendrite connections between neurons. In this case, the network's graph *G*_*S*_ is a directed multigraph, with the direction of an edge determined by the axon's origin. Signals propagating through the axon–dendrite network generate correlations between ROs in neurons. These correlations play a crucial role in constructing the QL representation of classical neuronal dynamics. Generally, the structure of connections between node-circuits is very complex and not limited to the axon–dendrite network (see the article for detailed analysis); some connections are established at the molecular level or via electromagnetic fields. The construction of the complete connection graph *G*_*S*_ is difficult, if not impossible. Moreover, for real neuronal networks in the brain and body, *G*_*S*_ is a hypergraph, and its structure varies with time. (We recall that in a hypergraph, edges connect clusters of nodes rather than individual nodes.) In our modeling, we do not employ this extremely complex geometry of connections within S and instead represent the network state by considering correlations between ROs in node-circuits (but cf. with Scholes, 2024; Amati and Scholes, 2025; Khrennikov et al., 2025b, where the graph geometry was explored). Thus, instead of the very complex hypergraph *G*_*S*_ of electrochemical connections between node-circuits, it is useful to represent *S* by the graph *G*_*S, cor*_ of correlations between ROs generated in the nodes of *G*_*S*_. This approach leads to the QL representation. The set of its nodes coincides with the nodes of the “electrochemical graph” *G*_*S*_, while its edges are determined by non-zero correlations between nodes: if the correlation between ROs in node-circuits 𝔰_*j*_ and 𝔰_*i*_ is non-zero, these nodes are connected by an edge. Such edges are undirected. Hence, *G*_*S*_ is a directed graph, but *G*_*S, cor*_ is an undirected graph.

The canonical basis in the linear space H consists of the vectors |1〉 = (10…0), …, |*N*〉 = (0…1). Any vector *v* ∈H can be expanded with respect to this basis, $v=\sum_{j}^{N}v_{j}|j\rangle .$ The basis vectors $(|j\rangle {)}_{j=1}^{N}$ represent the node-circuits of the network *S*. The node-circuits of S are represented by orthogonal vectors with respect to the scalar product. This orthogonality is a constraint on the model and, in principle, can be omitted.

This is the place to remark that the representation of network nodes by vectors in complex Hilbert space is a formal mathematical construction—the node-circuits are classical (macroscopic) neuronal structures. The networks under consideration are not quantum networks; they are classical networks for which we construct the QL representation.

### 3.1 Coupling between covariance matrices for neural oscillations and density matrices

Let vector *z* = *z*(ω) ∈ H be a random vector representing ROs generated by the neuronal network *S*. We proceed under the assumption that it has a zero mean value, μ = *E*[*z*] = 0. If this is not the case, one can always use the vector (*z*−μ), which has zero mean value. Consider the covariance matrix of this random vector:


$$\begin{array}{l} C=\left(c_{km}\right),c_{km}=E\left[z_{k}{\bar{z}}_{m}\right]. \end{array}$$ (2)

This is the matrix representation of the correlation graph *G*_*S, cor*_. It is a Hermitian and positive-definite matrix. Its only difference from a density matrix is that *C* may have a non-unit trace. It is natural to connect the classical ROs in *S* with quantum formalism, constructing a QL representation of the functioning of *S* via trace normalization (see Khrennikov, 2005, 2006a, 2014a, 2024a),


$$\begin{array}{l} C\;\to \;\rho =C/TrC. \end{array}$$ (3)

Such a density matrix is a QL state generated by ROs in *S*.

We speak of matrices to couple QLM to the neuronal basis. We can proceed in the basis-invariant framework with covariance and density operators. Thus, we refer to operators or their matrices. As in quantum theory, various bases in H can be employed. Bases other than the canonical node basis contain linear combinations of node state vectors, so such states are “non-local” from the perspective of brain geometry, as is the body in three-dimensional physical space. This reflects the non-locality of information processing.

The correspondence, ROs → covariance matrix, is not one-to-one; a variety of ROs generate the same *C*. Moreover, the correspondence *C* → ρ is also not one-to-one because of normalization scaling. Hence, QLM provides a fuzzy picture of classical random processes in a network.^2^

### 3.2 Generation of pure states

Now, consider the covariance matrix *C*_*j*_, such that only one element *c*_*jj*_ ≠ 0. It expresses ROs in *S* such that all circuits besides 𝔰_*j*_, are inactive (frozen). (For *i* ≠ *j*, condition $c_{ii}=E\left[|z_{i}{|}^{2}\right]=0$ implies that the random variable *z*_*i*_ = 0 almost everywhere.) While this is an idealized situation, it remains useful in a mathematical model. The corresponding density matrix represents the projection on the vector |*j*〉, ρ_*j*_ = *C*_*j*_/*c*_*jj*_ = |*j*〉〈*j*|. In principle, in this way, the circuit-basis vector |*j*〉 can be physically generated by activating a single circuit in isolation from others.

Now consider ROs with the covariance matrix *C*_*v*_ = (*c*_*ij*_ = *v*_*i*_*v*_*j*_), where vector *v* =(*v*_1_, …, *v*_*N*_) ∈ H. Then *C*_*v*_ = |*v*〉〈*v*| and ρ_*v*_ = |ψ_*v*_〉〈ψ_*v*_|, where ψ_*v*_ = *v*/||*v*||. Thus, such ROs generate pure states of this QLM. Due to the degeneration of correspondence, ROs → covariance (density) matrix, each pure state can be generated by a variety of ROs. What is common between such ROs? As was shown in Khrennikov (2005), Khrennikov (2006a), Khrennikov (2014a), and Khrennikov (2024a), each such random vector *z* = *z*(ω) is concentrated in the one-dimensional subspace *L*_ψ_ = {*v* = *c*|ψ_*v*_〉}. If vector *v* is a non-trivial superposition of the node-basis vectors (|*i*〉), that is $v=\sum_{i}v_{i}|i\rangle ,$ then ROs generating the QL state ρ_*v*_ are non-locally distributed; all neuronal nodes |*i*〉 with *v*_*i*_ ≠ 0 are involved in its generation.

We note that one of the ways to generate a pure state is to consider deterministic (non-random) dynamics in *S, z*_*t*_ with *z*_0_ = |*v*〉, where |*v*〉 is normalized to one (see Section 5). If this initial vector |*v*〉 is the eigenvector of QL Hamiltonian, then ρ_*v*_(*t*) ≡ |*v*〉〈*v*|. Thus, stationary pure states, one-dimensional projections, can be generated as Eigen states of Hamiltonian dynamics in *S* (see Section 5).

### 3.3 Ensemble vs. time averages

This is a good place to make the following theoretical remark on the mathematical description of correlations. In the classical probability model (Kolmogorov, 1933), the elements of covariance matrix (Equation 2) are calculated as the integrals


$$\begin{array}{l} c_{km}=\int_{\Omega }\;z_{k}\left(\omega \right){\bar{z}}_{m}\left(\omega \right)dP\left(\omega \right), \end{array}$$ (4)

where Ω is a sample space (Kolmogorov, 1933); its points are random parameters or elementary events) and *P* is the probability measure on Ω.

However, in experimental research (both in physics and biology) the following time averages are used. For two complex-valued time series *x*(*t*) and *y*(*t*), the covariance is defined as:


$$\begin{array}{l} Cov\left(x,y\right)=\frac{1}{T}\overset{T}{\underset{t=1}{\sum }}x\left(t\right)\bar{y}\left(t\right), \end{array}$$ (5)

where *T* is the total number of time points (we proceed under the assumption of zero mean values).

The coincidence of ensemble and time averages is a subtle mathematical issue; their equivalence relies on the assumption of ergodicity. This assumption is widely accepted and often applied automatically. In theoretical models, one typically works within the framework of Kolmogorov probability theory, whereas in experimental studies, time averages are generally used. However, the validity of the ergodicity hypothesis in the context of quantum and QL processes remains an open question (Kolmogorov, 1956; Shor et al., 2021a). A detailed discussion of this issue falls beyond the scope of the present article, and we shall not pursue it further here.

The Equation 5 raises the question of the selection of a proper time scale for averaging—that is, the selection of the parameter *T*. In neuroscience, this issue has been discussed, e.g., in Khrennikov (2017), Thomson (1982), Percival and Walden (1993), and Mitra and Pesaran (1999).

## 4 Classical vs. quantum realizations of observables on neuronal networks

Let *S* be a network with *N* neuronal circuits. ROs in *S* are represented by a classical random variable *z* = *z*(ω) valued in complex Hilbert space H of dimension *N*. (Here parameter ω describes randomness in *S*.)

Consider now a quadratic form *Q*_*A*_(*z*) = 〈*Az*|*z*〉 on H, where *A* is a Hermitian matrix. For a random vector *z* valued in H, we can consider the average of this form,


$$\begin{array}{l} E_{z}\left[Q_{A}\right]=\int_{\Omega }\;\langle Az\left(\omega \right)|z\left(\omega \right)\;\rangle \text{dP}\left(\omega \right)=\int_{H}\langle Aw|w\rangle dp_{z}\left(w\right), \end{array}$$ (6)

where Ω is a set of random parameters (elementary events), *P* is the probability measure, and *p*_*z*_ is the probability distribution of the H-valued random vector *z* = *z*(ω).

This average can be coupled to the covariance matrix by the following equality:


$$\begin{array}{l} E_{z}\left[Q_{A}\right]=TrCA. \end{array}$$ (7)

The right-hand side of this equality is (up to normalization) the quantum formula for calculation of averages of observables that are mathematically represented by Hermitian matrices (operators). In terms of the corresponding density matrix,


$$\begin{array}{l} \rho =\rho_{C}=C/Tr\;C \end{array}$$

we have


$$\begin{array}{l} {\langle A\rangle }_{\rho }=Tr\rho A=E_{z}\left[Q_{A}\right]/TrC\\ \;=\int_{H}\langle Aw|w\rangle dp_{z}\left(w\right)/\int_{H}\langle w|w\rangle dp_{z}\left(\text{w}\right). \end{array}$$ (8)

This formula couples the average 〈*A*〉_ρ_ of quantum observable *A* in the state ρ with the average of the corresponding quadratic form of ROs in the neuronal network *S*.

The correspondence rule


$$\begin{array}{l} A↔Q_{A} \end{array}$$ (9)

generates matching of quantum and classical averages. One can investigate this coupling in more detail and obtain from the quadratic form *Q*_*A*_ = *Q*_*A*_(ω) a discrete random variable with values *a*_*i*_, where (*a*_*i*_) is the spectrum of the operator *A*. Such a discrete random variable is obtained via the threshold detection scheme; the Born rule appears as an approximation of classical probability. This is a mathematically advanced formalism that cannot be presented here (see Cohen, 2014, for rigorous mathematical representation). In this model of classical-quantum coupling, the discretization procedure (threshold detection for quadratic forms of classical neuronal variables) is the source of “quantumness.” The phase space representation (Section 5) is purely classical. All quadratic forms are jointly defined as random variables on the same probability space. However, each threshold detection measurement procedure determines its own probability space. Generally, the corresponding discrete-valued observables cannot be represented as random variables on the same probability space. They may not be jointly measurable, as their measurement procedures need not be compatible. In this model, the appearance of incompatible observables originates from the transition from classical observables, given by quadratic forms, to QL observables, mathematically represented by Hermitian operators.

Consider now a dichotomous observable *A* yielding values 0, 1. It has two representations, classical and QL. In the QL representation, *A* = *E* is given by a projector on subspace *L*; in the classical representation, *A* is given by the quadratic form *Q*_*E*_. Let ROs in *S* are described by the random vector *z* with the covariance matrix *C*, the QL state ρ = *C*/Tr *C*. Since *A*'s average is equal to the probability of the outcome *A* = 1, we have the following coupling between this probability and classical average of *Q*_*E*_,


$$\begin{array}{l} P\left(A=1|\rho \right)=E_{z}\left[Q_{E}\right]/TrC\\ \;=\int_{H}\langle Ew|w\rangle dp_{z}\left(w\right)/\int_{H}\langle w|w\rangle dp_{z}\left(w\right). \end{array}$$ (10)

It can be interpreted as the “weight” of ROs in the subspace *L* = *E* H relatively to the weight of ROs the whole space H. Thus, this formula connects the outcomes of observations over a neuronal network *S* with averaging of ROs in it.

Generally, if $A=\underset{i}{\sum }a_{i}E_{a_{i}},$ where (*E*_*a*_*i*__) is the spectral family of the operator *A*, then we have


$$\begin{array}{l} P\left(A=a_{i}|\rho \right)=E_{z}\left[Q_{E_{a_{i}}}\right]/TrC. \end{array}$$ (11)

If operator *A* has non-degenerate spectrum with eigenvectors (|*a*_*i*_〉), then


$$\begin{array}{l} P\left(A=a_{i}|\rho \right)=c_{ii}/\underset{j}{\sum }c_{jj}, \end{array}$$ (12)

where (*c*_*jj*_) are diagonal elements of the covariance matrix *C* in the basis (|*a*_*i*_〉). Hence, the probabilities of outcomes are straightforwardly coupled with the elements of the covariance matrix.

Let *A*_1_ and *A*_2_ be two compatible observables that is they can be jointly measurable. In the QL representation, they are described as commuting Hermitian operators *A*_1_ and *A*_2_. Their quantum correlation is given by the formula


$$\begin{array}{l} {\langle A_{1}A_{2}\rangle }_{\rho }=Tr\rho \;A_{1}A_{2}=Tr\rho \;A_{2}A_{1}. \end{array}$$ (13)

So, in fact, this is the average of the observable *A* described by the (Hermitian) operator *A* = *A*_1_*A*_2_ = *A*_2_*A*_1_. By applying correspondence rule (Equation 9) to this observable, we obtain the classical average representation of quantum correlations


$$\begin{array}{l} Tr\rho \;A_{1}A_{2}=E_{z}\left[Q_{A_{1}A_{2}}\right]/TrC\\ \;=\int_{H}\langle \;A_{1}A_{2}w|w\rangle dp_{z}\left(w\right)/\int_{H}\langle w|w\rangle dp_{z}\left(w\right). \end{array}$$ (14)

This possibility of a classical representation of quantum correlations may appear to contradict the violation of Bell inequalities. However, it has been shown that this is not the case (Khrennikov, 2014a). Bell-type inequalities can, in principle, be tested for neuronal networks in the brain (as well as for other types of networks, such as social networks). We examined this for observables determined by EEG measurements in article (Shor et al., 2021a).

On the classical phase space, one can consider not only quadratic forms but also arbitrary functions, *z* → *f*(*z*). Can one identify their QL images? In Khrennikov (2005), Khrennikov (2006a), Khrennikov (2014a), and Khrennikov (2024a), the following coupling between classical (functional) and QL (operator) descriptions was considered: $f\to \frac{f^{\prime \prime }(0)}{2}$ for a twice differentiable function *f* = *f*(*z*).

## 5 Coupling dynamics of coupled harmonic oscillators and the Schrödinger dynamics of QL states

PCSFT provides a classical foundation for quantum mechanics (Khrennikov, 2005, 2006a, 2014a, 2024a). The central idea is that the Schrödinger equation, which lies at the heart of quantum theory, can be derived from or coupled with *Hamiltonian dynamics* on a classical phase space. This framework examines the quantum–classical interface from both mathematical and conceptual perspectives. At its core, PCSFT interprets quantum states as labels for statistical ensembles of coupled classical oscillators. We use this formalism to couple the patterns generated by neuronal networks, mathematically represented as systems of coupled harmonic oscillators, and the dynamics of QL cognitive states.

For *N* classical oscillators, the phase space Φ = *Q* × *P* = ℝ^*N*^ × ℝ^*N*^. This corresponds to a real Hilbert space with the scalar product (ϕ_1_|ϕ_2_) = (*q*_1_|*q*_2_) + (*p*_1_|*p*_2_), where ϕ_*i*_ = {*q*_*i*_, *p*_*i*_} ∈ Φ. This phase space underpins quantum mechanics with a finite-dimensional state space, the complex Hilbert space H = ℂ^*N*^, endowed with the scalar product.

Quantum mechanics on physical space ℝ^3^ is based on the infinite-dimensional Hilbert space of square-integrable complex-valued functions, essentially the Hilbert space H = *L*^2^(ℝ^3^, ℂ). The underlying classical phase space is given by Φ = *L*^2^(ℝ^3^, ℝ) × *L*^2^(ℝ^3^, ℝ). The real and imaginary parts of a wavefunction |ψ〉 ε H correspond to coordinates and momenta in an infinite-dimensional phase space Φ. But we restrict our considerations to finite dimensional systems.

Any phase space can be endowed with a *symplectic structure*. In this setting, a symplectic form ω is introduced as:


$$\begin{array}{l} \omega \left(\phi_{1}|\phi_{2}\right)=\left(\phi_{1}|J\phi_{2}\right), \end{array}$$ (15)

where *J* is the symplectic structure operator defined as $J=|\begin{array}{cc} 0 & I_{p}\\ -I_{q} & 0 \end{array}|,$ where *I*_*q*_, *I*_*p*_ are the unit operators in *Q, P*. In the complex Hilbert space, H corresponds to the phase space Φ and the operator *J* is represented as the operator of multiplication by *i* (we remark that H is complexification of Φ, namely, H = *Q* ⊕ *iP*).

The Hamiltonian functional *H*(ϕ) generates dynamics via


$$\begin{array}{l} \dot{\phi }\left(t\right)=J\nabla H\left(\phi \left(t\right)\right). \end{array}$$

When the Hamiltonian functional is *quadratic*, that is, given by a symplectically invariant quadratic form *H*(ϕ) = (ϕ|*Hφ*), the evolution reduces to the linear Schrödinger equation:


$$\begin{array}{l} i\frac{d\psi }{dt}\left(t\right)=H\psi \left(t\right) \end{array}$$ (16)

Thus, the Schrdinger equation appears not as a postulate, but as a dynamical law for classical phase space dynamics under a symplectic structure.

In PCSFT, a quantum state (wavefunction or density operator) corresponds to the covariance operator of classical oscillations. Quantum averages emerge from classical statistical averages over this ensemble, allowing an interpretation in which quantum randomness is epistemic—arising from incomplete knowledge of underlying classical oscillations. In this way, quantum mechanics can be understood as a *projection or statistical encoding* of a deeper classical theory, with the Schrödinger equation derived from the Hamiltonian flow of an underlying symplectic system.

We now briefly present the above consideration with *q, p* coordinates. Any Hamiltonian function *H*(*q, p*) generates the system of Hamiltonian equations


$$\begin{array}{l} \dot{q}=\frac{\partial H}{\partial p}\left(q,p\right),ṗ=-\frac{\partial H}{\partial q}\left(q,p\right). \end{array}$$

Consider now a quadratic and symplectically invariant Hamiltonian function


$$\begin{array}{l} H\left(q,p\right)=1/2\left[\left(Rp,p\right)+2\left(Tp,q\right)+\left(Rq,q\right)\right],\; \end{array}$$

where *R* is a symmetric operator, *R*^⋆^ = *R* and *T*^⋆^ = −*T*. The operator (the Hessian of the Hamilton function) is given as


$$\begin{array}{l} H=|\begin{array}{cc} R & \;T\\ -T & \;R \end{array}|, \end{array}$$

which commutes with the symplectic operator *J*. This is a system of harmonic oscillators, and it can be rewritten as the Schrödinger equation.

Here *I*_*q*_, *I*_*p*_ are the unit operators in *Q, P*. In the complex Hilbert space, H corresponds to the phase space Φ and the operator *J* is represented as the operator of multiplication by *i* (we remark that H is complexification of Φ, namely, H = *Q* ⊕ *iP*).

The Hamiltonian functional *H*(ϕ) generates dynamics via


$$\begin{array}{l} \dot{\phi }\left(t\right)=J\nabla H\left(\phi \left(t\right)\right). \end{array}$$

When the Hamiltonian functional is *quadratic*, that is, given by a symplectically invariant quadratic form *H*(ϕ) = (ϕ|*Hφ*), the evolution reduces to the linear Schrödinger equation:


$$\begin{array}{l} i\frac{d\psi }{dt}\left(t\right)=H\psi \left(t\right) \end{array}$$

Thus, the Schrödinger equation appears not as a postulate, but as a dynamical law for classical phase space dynamics under a symplectic structure. We remark that in this framework, ψ(*t*)is complexification of the phase space variable ϕ(*t*): *q(t)* = *Re* ψ(*t*) and *p(t)*= *Im* ψ(*t* ).

In PCSFT, a quantum state (wavefunction or density operator) corresponds to the covariance operator of classical oscillations. Quantum averages emerge from classical statistical averages over this ensemble, allowing an interpretation in which quantum randomness is epistemic—arising from incomplete knowledge of underlying classical oscillations. In this way, quantum mechanics can be understood as a *projection or statistical encoding* of a deeper classical theory, with the Schrödinger equation derived from the Hamiltonian flow of an underlying symplectic system.

We now briefly present the above consideration with *q, p* coordinates. Any Hamiltonian function *H*(*q, p*) generates the system of Hamiltonian equations


$$\begin{array}{l} \dot{q}=\frac{\partial H}{\partial p}\left(q,p\right),ṗ=-\frac{\partial H}{\partial q}\left(q,p\right).\; \end{array}$$

Consider now a quadratic and symplectically invariant Hamiltonian function


$$\begin{array}{l} H\left(q,p\right)=1/2\left[\left(Rp,p\right)+2\left(Tp,q\right)+\left(Rq,q\right)\right],\; \end{array}$$

where *R* is a symmetric operator, *R*^⋆^ = *R* and *T*^⋆^ = −*T*. The operator (the Hessian of the Hamilton functions) $H=|\begin{array}{cc} R & \;T\\ -T & \;R \end{array}|,$

which commutes with the symplectic operator *J*. This is a system of harmonic oscillators, and it can be rewritten as the Schrödinger equation.

We now consider an illustrative example of interconnection of Hamiltonian dynamics, dynamics of a system of coupled harmonic oscillators, and QL state dynamics driven by the Schrödinger equation.

**Hamiltonian function and equations:** We take the quadratic Hamiltonian functional with matrices


$$\begin{array}{l} R=\left(\begin{array}{cc} 1 & 0\\ 0 & 4 \end{array}\right),R=\left(\begin{array}{cc} 0 & -0.5\\ 0.5 & 0 \end{array}\right),T^{*}=-T. \end{array}$$

The Hamilton equations can be written as a system of first order ordinary differential equations for the variable *x* = (*q,p*) ε R^4^,


$$\begin{array}{l} \dot{x}=Ax,A=\left(\begin{array}{cc} -T & R\\ -R & -T \end{array}\right)=\left(\begin{array}{cccc} 0 & 0.5 & 1 & 0\\ -0.5 & 0 & 0 & 4\\ -1 & 0 & 0 & 0.5\\ 0 & -4 & -0.5 & 0 \end{array}\right) \end{array}$$

Eigenvalues of *A* are purely imaginary:


$$\begin{array}{l} \lambda =\left\{\pm 0.918861i,\;\pm 4.081139i\right\}, \end{array}$$

corresponding to two oscillation frequencies ω_1_ ≃ 0.9189, ω_2_ ≃ 4.0811.

**Second-Order System for** ***q:*** Eliminating *p* gives


$$\begin{array}{l} \ddot{q}+C\dot{q}+Kq=\;0, \end{array}$$

where


$$\begin{array}{l} \begin{array}{c} C=T+RTR^{-1}=\left(\begin{array}{cc} 0 & -0.625\\ 2.5 & 0 \end{array}\right)\\ K=R^{2}+RTR^{-1}T=\left(\begin{array}{cc} 0.9375 & 0\\ 0 & 15 \end{array}\right) \end{array} \end{array}$$

Thus, the system represents two *coupled harmonic oscillators* (via the velocity-coupling matrix *C*).

**Schrödinger representation:** The corresponding complex Schrödinger equation has Hamitonian:


$$\begin{array}{l} H=\left(\begin{array}{cc} R & T\\ -T & R \end{array}\right)\left(\begin{array}{cccc} 1 & 0 & 0 & -0.5\\ 0 & 4 & 0.5 & 0\\ 0 & 0.5 & 1 & 0\\ -0.5 & 0 & 0 & 4 \end{array}\right) \end{array}$$

Eigenvalues of *H* are identical to the positive frequencies: *E* = {0.918861, 4.081139}. The solution is ψ(*t*) = *e*^−*itH*^ψ(0). This shows that the linear Schrödinger equation emerges naturally from the classical Hamiltonian flow under a symplectic structure.

**Figure 2:**
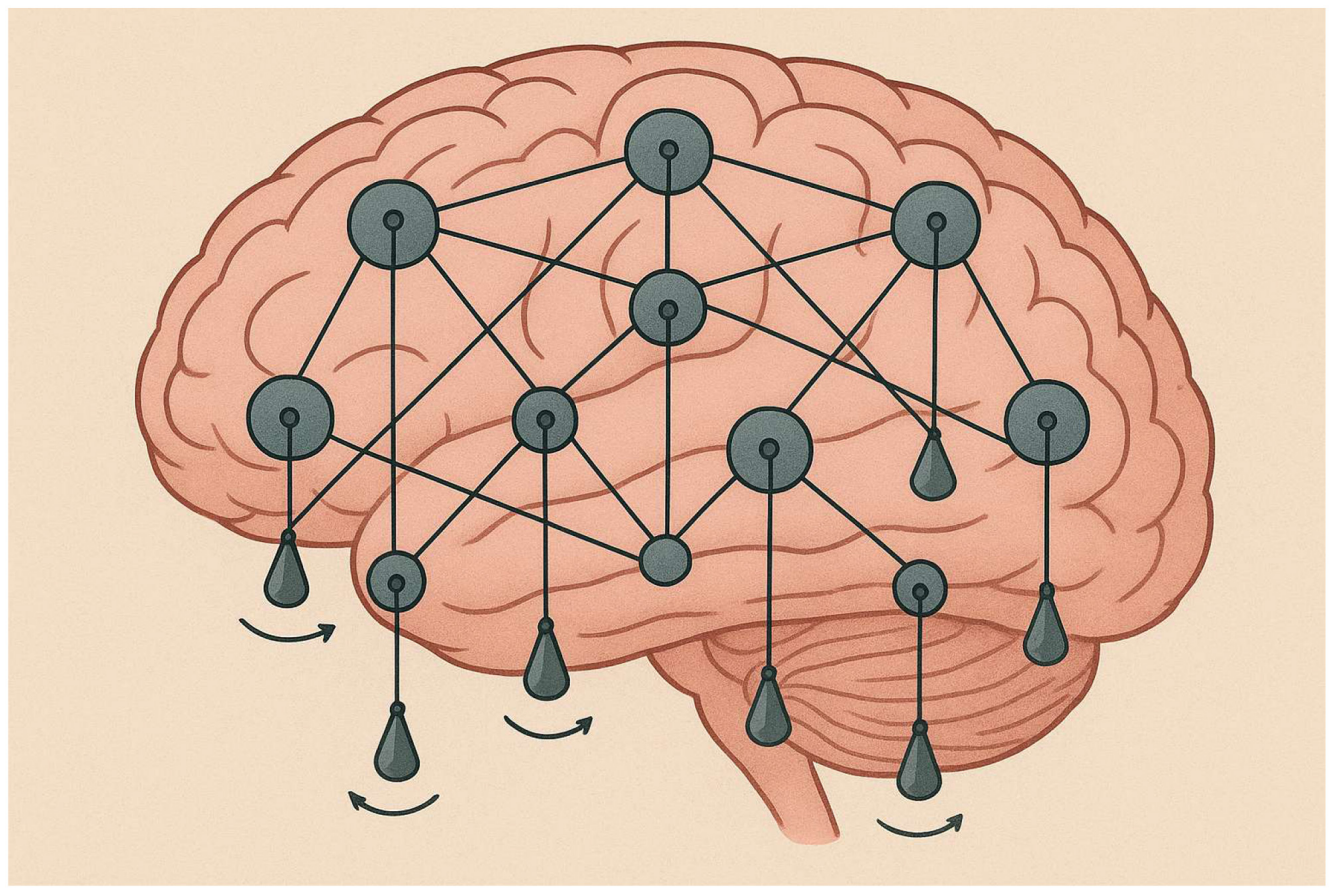
Oscillatory brain: a network of pendulum-like coupled oscillators.

**Figure 3:**
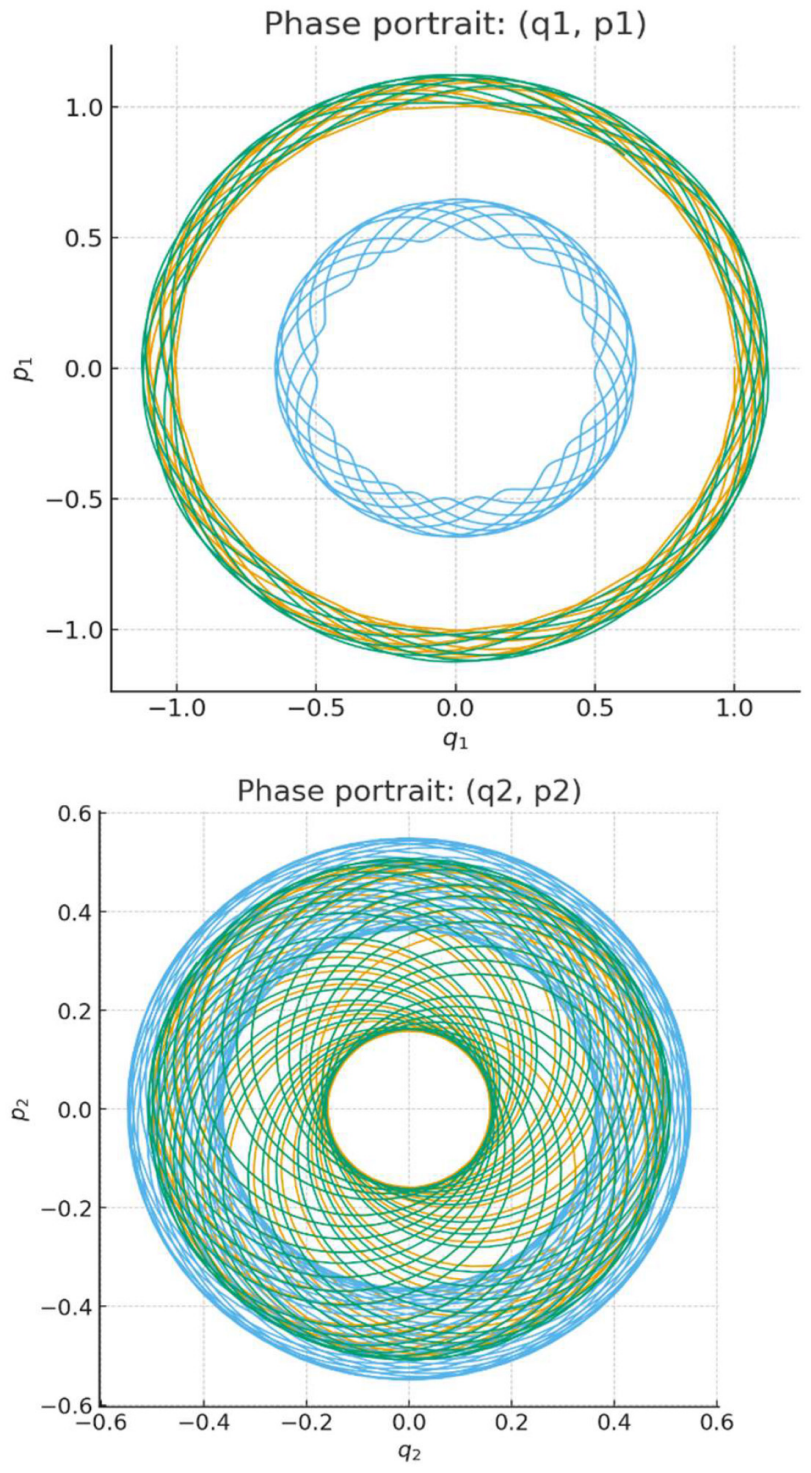
**(a, b)** The phase space trajectories for the system of coupled harmonic oscillators under consideration. The same portraits are generated for real plane representations of two (complex) coordinates of solutions of the corresponding Schrödinger equation.

Numerical simulation of solutions, ψ*(t)* = *(*ψ_1_*(t)*, ψ_2_*(t))*, of this Schrödinger equation (without the normalization constraint on the initial conditions) reproduces Figures 2, 3, where Figures 3a, b correspond now to graphs (Re ψ_1_, Im ψ_1_) and (Re ψ_2_, Im ψ_2_), respectively.

Finally, we remark that by considering random initial conditions, we generate two stochastic processes valued in phase space: ϕ(*t*) = *(q(t),p(t))* ε Φ and in complex Hilbert space: ψ*(t)* ε H. The latter stochastic process is complexification of the former. Consider now, for any *t*, the covariance operator *C(t)* of the Hilbert space valued random variable ψ*(t) and* proceed under the normalization constraint: *Tr C(0)* = 1. This trace normalization is preserved in the process of evolutio*n, Tr C(t)* = 1. So, this covariance matrix is a density matrix: ρ*(t)* = *C(t)* describing mathematically a mixed QL-state at the instance of time *t*. It satisfies the *von Neumann equation* for the evolution of density operators in quantum mechanics.

## 6 Entanglement of observables

In this Section, we present the observational viewpoint on entanglement (see (Zanardi, 2001); (Zanardi et al., 2004); (Basieva and Khrennikov, 2022); (Khrennikov and Basieva, 2023). It will be employed in our QLM in Section 7. The traditional approach, viewing entanglement as the correlation of the states of two systems (Werner, 1989), in our case, two neuronal networks *S*_1_ and *S*_2_, will be employed in Section 8.

We begin with a simple example that illustrates the general construction to be developed later.

Let dim H = 4, let commuting Hermitian operators *A*_1_ and *A*_2_ have eigenvalues *a*_1_ = ±1, *a*_2_ = ±1, each with degeneracy 2. Consider the basis (|*ij*〉), *i, j* = ±, in H consisting of the joint eigenvectors, *A*_1_|*ij*〉 = *i*|*ij*〉, *A*_2_|*ij*〉 = *j*|*ij*〉. Any vector in H can be expanded w.r.t. this basis,


$$\begin{array}{l} |\psi \rangle =\sum_{ij}w_{ij}|ij\rangle . \end{array}$$ (17)

Such vector decomposition generates on H the structure of tensor product H_1_ ⊗ H_2_, where H_1_ and H_1_ are two-dimensional Hilbert spaces with the bases (|*i*〉_1_, *i* = ±), and (|*i*〉_2_, *i* = ±); vector


$$\begin{array}{l} |\phi \rangle =\sum_{ij}w_{ij}|i\rangle_{1}⊗|j\rangle_{2} \end{array}$$ (18)

is identified with vector |ψ〉 given by Equation 17. Tensor product on H_1_ ⊗ H_2_ induces tensor-product structure on H.

Consider now complex Hilbert space *dim* H = *N* = *N*_1_*N*_2_. Let commuting Hermitian operators *A*_1_ and *A*_2_ have eigenvalues (*a*_1*j*_), *j* = 1, …, *N*_1_, (*a*_2*i*_), *i* = 1, …, *N*_2_. Each *a*_1*j*_ has degeneracy *N*_2_ and each *a*_2*j*_ has degeneracy *N*_1_. Consider the basis (|*ij*〉 ≡ |*a*_1*i*_*a*_2*j*_〉), *i*1, …, *N*_1_, *j* = 1, …, *N*_2_ in H consisting of their joint eigenvectors, *A*_1_|*ij*〉 = *a*_1*i*_|*ij*〉, *A*_2_|*ij*〉 = *a*_2*j*_|*ij*〉. Any vector in H can be expanded w.r.t. this basis, see ([any]). Such vector decomposition generates on H the structure of tensor product H_1_ ⊗ H_2_, where H_1_ and H_2_ are Hilbert spaces of the dimensions *N*_1_ and *N*_2_ with the bases (|*i*〉_1_) and (|*j*〉_2_), and the isomorphism map *T:* H_1_ ⊗ H_2_→H is determined by its action on the basis vectors |*i*〉_1_ ⊗ |*j*〉_2_ → |*ij*〉.

If the coefficients in representation (Equation 17) can be factorized, $w_{ij}=w_{i}^{\left(1\right)}w_{j}^{\left(2\right)},$ then formally vector |ψ〉 belonging to H can be written as


$$\begin{array}{l} |\psi \rangle =\left(\sum_{i}w_{i}^{\left(1\right)}|i\rangle_{1}\right)⊗\left(\sum_{j}w_{j}^{\left(2\right)}|j\rangle_{2}\right). \end{array}$$ (19)

Such a vector is called separable; otherwise, |ψ〉 is called entangled. We remark that if |ψ〉 = *T*(|ϕ_1_〉 ⊗ |ϕ_2_〉), then it is factorizable. Thus, the notions of separability vs. entanglement given in Equation 18 are equivalent to the usual notions of separability vs. entanglement in the tensor product of Hilbert spaces.

Consider now spaces of density operators *D*(H_1_), *D*(H_2_), *D*(H), then *D*(H) is isomorphic to tensor product *D*(H_1_) ⊗ *D*(H_2_). The notions of separability vs. entanglement for density operators (mixed states) is transferred to the space *D*(H).

The symbol *L(M)* is denoted by the space of linear operators acting in a complex Hilbert space *M*. We recall that we consider the finite-dimensional case.

In Hilbert space H_1_ ⊗ H_2_ consider two operator algebras:


$$\begin{array}{l} {\text{A}}_{1}=\left\{A_{1}=a_{1}⊗I:a_{1}\in L\left(H_{1}\right)\right\},\\ {\text{A}}_{2}=\left\{A_{2}=I⊗\;a_{2}:a_{2}\in L\left(H_{2}\right)\right\}. \end{array}$$ (20)

Hermitian operators belonging to these algebras are called “local observables.” For our neurophysiological applications, it is important to note that this is a tensor-product locality; generally, it has nothing to do with space-time locality. The images of these algebras in *L*(H) are denoted as **A**_**i**_(H), *i* = 1,2, or simply **A**_**i**_. These local algebras induce the structure of the tensor product of operators in H; for *A*_*i*_ ∈ **A**_**i**_(H), *i* = 1,2, we set *A*_1_ ⊗ *A*_2_ = *A*_1_°*A*_2_ = *A*_2_°*A*_1_. We remark that if *A*_1_ ∈ **A**_**1**_(H), *A*_2_ ∈ **A**_**2**_(H), then [*A*_1_, *A*_2_] = 0; so Hermitian operators from algebras **A**_**1**_(H) and **A**_**2**_(H) represent compatible observables.

To clarify the meaning of entanglement (which up to now has been treated from a mathematical viewpoint), consider the four-dimensional case and the singlet state


$$\begin{array}{l} |\psi \rangle =(|+\rangle |-\rangle -|-\rangle |+\rangle )/\sqrt{2}. \end{array}$$ (21)

It is entangled according to the above mathematical definition. But what does it mean physically and biologically (Sections 7 and 8)? As noted, this formula encodes correlations between the outcomes of two observables *A*_1_ and *A*_2_: the conditional probabilities *P*(*A*_2_ = ±|*A*_1_ = ∓) = 1 as well as *P*(*A*_1_ = ±|*A*_2_ = ∓) = 1. These correlations, for each pair of such observables, are purely classical. “Quantumness” appears because of the existence of *incompatible observables*, *A*_*i*_, *B*_*i*_ ∈ **A**_**i**_(H) such that [*A*_*i*_, *B*_*i*_] ≠ 0, *i* = 1, 2. Here, non-commutativity expresses the impossibility of joint measurements of two observables. The singlet state can also be represented as


$$\begin{array}{l} |\psi \rangle =(|C=+\rangle |D=-\rangle -|C=-\rangle |D=+\rangle )/\sqrt{2}, \end{array}$$ (22)

where *C* = *A*_1_, *D* = *A*_2_ or *C* = *B*_1_, *D* = *B*_2_ and the operators *B*_*i*_ can be selected as non-commuting with *A*_*i*_, *i* = 1, 2. Thus, this entangled state encodes correlations between families of local observables that are jointly non-measurable. Classical probability describes only correlations for families of jointly measurable observables. This represents the incompatibility (non-commutativity) interpretation of entanglement.

## 7 Entanglement of observables on neuronal circuits

Here, we employ the observational viewpoint on entanglement presented in Section 6. Let *S* be a network with *N* = *N*_1_*N*_2_ neuronal circuits. Let *A*_1_ and *A*_2_ be observables on *S* as in Section 6. The only new component is that these QL observables are coupled to the network as the QL images of the corresponding quadratic forms *Q*_*A*_1__ and *Q*_*A*_2__ of ROs in *S*. Neuronal node-circuits 𝔰_*i*_, *i* = 1, …, *N*, are renumerated as 𝔰_*ij*_, *i* = 1, …, *N*_1_, *j* = 1, …, *N*_2_. The biological counterpart of this mathematical construction is that, for each node-circuit, both observables *A*_1_ and *A*_2_ can be jointly measured, as well as any two observables from the operator algebras **A**_**1**_ and **A**_**2**_.

If a circuit 𝔰 is not activated, then in the classical representation *z*_𝔰_ = 0 a.e., where *z*_𝔰_ is the random variable describing ROs in 𝔰.

Consider one node-circuit 𝔰 = 𝔰_*ij*_. Let only this circuit be activated in the network *S*. In the classical representation, all random variables *z*_𝔰_ = 0 a.e. for 𝔰 ≠ 𝔰_*k*_ and $E\left[|z_{k}{|}^{2}\right]\neq 0,$ where we set *z*_*k*_ ≡ *z*_𝔰_*k*__. In the QL representation, *S* is in the pure state |*k*〉 = |*ij*〉. A measurement of the observables *A*_1_ and *A*_2_ on the network *S* gives the outputs *A*_1_ = *a*_1*i*_ and *A*_2_ = *a*_2*i*_ with probability 1.

Let only two node-circuits be activated in *S*: 𝔰_*k*_ = 𝔰_*i*_1_*j*_1__, 𝔰_*m*_ = 𝔰_*i*_2_*j*_2__, that is, in the classical representation, *z*_*n*_ = 0 a.e. for *n* ≠ *k, m* and $E\left[|z_{k}{|}^{2}\right],E\left[|z_{m}{|}^{2}\right]\neq 0.$ Let ROs in 𝔰_*k*_, 𝔰_*m*_ be correlated, generating the covariance matrix *C* with the elements *c*_*kk*_ = 1, *c*_*mm*_ = 1, *c*_*km*_ = −1, *c*_*mk*_ = −1 and other elements in *C* equal to zero. Hence, there is perfect anti-correlation between ROs in circuits 𝔰_*k*_ and 𝔰_*m*_. The corresponding QL state ρ = *C*/2 = |ψ〉〈ψ|, where |ψ〉 is the singlet state (Equation 21).

Let *i*_1_ = +, *j*_1_ = −, *i*_2_ = −, *j*_2_ = +. The circuits 𝔰_+−_, 𝔰_−+_ generate ROs


$$\begin{array}{l} z=z_{+-}|+-\rangle -z_{-+}|-+\rangle , \end{array}$$

where *z*_+−_ = *z*_−+_ a.e. Thus, the singlet state (Equation 21) is the QL image of this random variable. Such correlation is purely classical and does not represent the essence of the QL framework. As was already noted in 4, the strength of employing the QL linear space representation lies in the possibility (for the brain) of using a variety of bases. We have considered the neuronal basis, but the same singlet state carries anti-correlations in various bases (see Equation 22), whose elements are not localized in specific neuronal circuits.

Formally (mathematically), the neuronal network S can be represented as a compound system *S* = (*S*_1_, *S*_2_) of two systems *S*_1_ and *S*_2_ with the state spaces H_1_ and H_2_. In this observational framework, these systems are not identified with specific neuronal networks. They are formally extracted from S with the aid of two algebras of commuting observables, **A**_1_ and **A**_2_. Measurements performed by observables belonging to **A**_*i*_ are formally treated as “local observables” for subsystem *S*_*i*_, *i* = 1, 2.^3^

The correlations of local observables can be represented as the QL-average. For *B*_1_ = *b*_1_⊗*I* and *B*_2_ = *I*⊗*b*_2_,


$$\begin{array}{l} {\langle b_{1}b_{2}\rangle }_{\rho }=Tr\rho \;b_{1}{\;⊗b}_{2},\rho =\frac{C}{Tr\;C}, \end{array}$$ (23)

where *C* is the covariance operator of ROs in *S*, which are represented by a random variable *z* valued in tensor product Hilbert space H = H_1_ ⊗ H_2_ and not in Cartesian product Hilbert space H_1_ ⊕ H_2_. As was pointed out in Section 3, such a correlation can be presented in the classical probabilistic framework as


$$\begin{array}{l} {\langle b_{1}b_{2}\rangle }_{\rho }\;=\frac{1}{Tr\;C}\;=\int_{H_{1}⊗H_{2}}\langle \;b_{1}{\;⊗b}_{2}\;w|w\rangle dp_{z}\left(w\right). \end{array}$$ (24)

Entanglement is the Hilbert space expression of correlations between local observables—from algebras **A**_**1**_(H) and **A**_**2**_(H). The crucial difference from the classical probabilistic representation is that *these algebras contain incompatible observables which cannot be jointly measurable*.

Finally, we stress once again that decomposition of a neuronal network *S* into subsystems, *S* = (*S*_1_, *S*_2_), is not physical. Subsystems are virtual, and they express biological functions of *S*, not its neuronal architecture. Decomposition is not unique; even dimensions of the components of the tensor product can vary for the same biophysical neuronal network *S*, say if *N* = 12, we can factorize it as *N*_1_ = 3, *N*_2_ = 4 or *N*_1_ = 2, *N*_2_ = 6.

## 8 Entanglement of neuronal states

We start with a recollection of the notion of entanglement for pure and mixed states.

A pure state |ψ〉 belonging to tensor product of two Hilbert spaces H = H_1_ ⊗ H_2_ is called separable if it can be factorized as


$$\begin{array}{l} |\psi \rangle ={|\psi \rangle }_{1}⊗\;{|\psi \rangle }_{2},where{|\psi \rangle }_{i}\in H_{i}, \end{array}$$ (25)

otherwise a pure state is called entangled. A mixed state given by a density operator ρ is called separable if it can be represented as a mixture


$$\begin{array}{l} \rho =\underset{k}{\sum }p_{k}\;\rho_{k1}⊗\rho_{k2},\;\left(25\right) \end{array}$$ (26)

where ρ_*ki*_ ε H_*i*_ and the weights (*p*_*k*_) form a probability distribution, $p_{k}>0,{\underset{k}{\sum }p}_{k}=1.$ A non-separable state is called entangled. For pure states, it is straightforward to decide whether a state is separable or not. For mixed states, it is very difficult.

Although these definitions are commonly accepted in quantum theory, a careful reading of the original works of Schrödinger (Khrennikov, 2014b) may give the impression that he discussed “entanglement of observable” (cf. with discussion in Khrennikov and Basieva, 2023).

Consider now two networks *S*_1_ and *S*_2_ consisting of neuronal circuits 𝔰_1, *j*_, *j* = 1, …, *N*_1_, and 𝔰_2, *j*_, *j* = 1, …, *N*_2_, respectively. As before, for simplicity, suppose that each circuit generates one complex dimension. So, in QLM for *S*_1_ and *S*_2_, the complex Hilbert spaces H_1_ and H_2_ have dimensions *N*_1_ and *N*_2_. ROs in them are mathematically represented as random vectors *z*_*i*_ ∈ H_*i*_, *i* = 1,2; here *z*_*i*_ = (*z*_*i*, 1_, …, *z*_*i*,_*N*__*i*__).

Consider now the network *S*_⊕_ consisting of node-circuits of *S*_1_ and *S*_2_ and its graph picturing. The set of nodes of the graph *G*_*S*_⊕__ is the *union* of the sets of nodes of the graphs *G*_*S*_1__ and *G*_*S*_2__; the set of its edges includes all edges of *G*_*S*_1__ and *G*_*S*_2__ as well as additional edges representing the connections between some nodes of *G*_*S*_1__ and *G*_*S*_2__. According to our approach, the edge structure of the graph *G*_*S*_⊕__ is not visible in the QL representation, which is instead based on the correlation graph *G*_*S*_1_⊕ *S*_2_; *cor*_. The edges of this graph correspond to non-zero correlations between ROs in the corresponding nodes.

In QLM for such a network, its complex Hilbert space H_*S*_⊕__ = H_1_ ⊕ H_2_ has the dimension (*N*_1_ + *N*_2_). ROs in *S*_⊕_ are mathematically represented the random vector **z** = (*z*_1_, *z*_2_) ∈ H_*S*_⊕__, so in the node-basis **z** = (*z*_1_ … *z*_*N*_1_ + *N*_2__). The covariance matrix of ROs has the dimension ${\left(N_{1}+N_{2}\right)}^{2}.$ This dimension does not match the dimension of a density matrix for a quantum compound system, that is $N^{2},N=N_{1}N_{2}.$ Such a network cannot be used for generating entanglement.

We suggest the following construction of a compound network *S*_⊗_ whose complex Hilbert space is not a direct sum but a tensor product, H_*S*_⊕__ = H_1_⊕H_2_, and the covariance matrix of ROs in *S*_⊗_ has the dimension *N*^2^. Creation of the network *S*_⊗_ that is able to generate entangled states is characterized by the emergence of new circuits not present (or activated) in *S*_⊕_.

Each pair of circuits 𝔰_1, *i*_ ∈ *S*_1_ and 𝔰_2, *j*_ ∈ *S*_2_ is combined into the new circuit 𝔰_*ij*_. How can such a compound circuit be created? Since 𝔰_*ij*_ consists of the same neurons as the circuits 𝔰_1, *i*_, 𝔰_2, *j*_ the only new structure in the circuit 𝔰_*ij*_ arises from generating (activation) new channels for communication between neurons in 𝔰_1, *i*_ and neurons in 𝔰_2;*j*_. These channels can be physical axon-dendrite connections activated in the network *S*_⊗_ (but not active before). Another testable hypothesis is that *entangling channels* are routes for electromagnetic signaling between neurons across the circuits 𝔰_1, *i*_ and 𝔰_2, *j*_ (Schrödinger, 1935; Buzsáki et al., 2012; Anastassiou et al., 2011; Fröhlich and McCormick, 2010). Chemical signaling may also contribute to the formation of the entanglement-generating network *S*_⊗_, albeit on slower time scales.

One can hypothesize that the brain can generate entangled networks using various communication channels to perform different tasks. Generally, the three sorts of communication channels mentioned can be involved in the creation of circuits 𝔰_*ij*_ ∈ *S*_⊗_; each such circuit is given by the triple


$$\begin{array}{l} {𝔰}_{ij}=\left({𝔰}_{1,i},e_{ij},{𝔰}_{2,j}\right), \end{array}$$ (27)

where *e*_*ij*_ denotes the signaling channel between the neuronal circuits 𝔰_1, *i*_ and 𝔰_2, *j*_. ROs in this circuit are described by a random variable *Z*_*ij*_ = *Z*_*ij*_(ω), where ω is a chance parameter. Such ROs generate the covariance matrix $C_{ij;km}=E\left[Z_{ij}{\bar{Z}}_{km}\right],$ whose elements are the correlations between circuits 𝔰_*ij*_ and 𝔰_*km*_. This matrix has the dimension ${\left(N_{1}N_{2}\right)}^{2}.$ In QLM, the corresponding density matrices are obtained via normalization by trace; in the operator representation, they act in the complex Hilbert space H of the dimension *N* = *N*_1_*N*_2_.

We remark that these compound circuits need not be connected at the physical level, for example, by an axon-dendrite connection. Signals propagating in channels *e*_*ij*_ and *e*_*km*_ generates electromagnetic fields, and they can be non-trivially correlated (see Section 9 on *ephaptic* generation of such correlations).

Thus, circuits 𝔰_*ij*_ are vertices of the graph *G*_*S*_1_⊗ *S*_2_; *cor*_; its edges *E*_*ij, km*_ connect these vertices and represent correlations between ROs in circuits 𝔰_*ij*_ and 𝔰_*km*_. We discuss only the graph *G*_*S*_1_⊗ *S*_2_; *cor*_, in which edges *E*_*ij, km*_ represent correlations between corresponding circuits 𝔰_*ij*_ and 𝔰_*km*_. The graph of physical connections is not essential for QLM.

What is about the tensor-product structure of the Hilbert space H? Let only one circuit 𝔰_*ij*_ be activated and let it generate ROs *Z*_*ij*_. In the corresponding (self-)covariance matrix *C*_*ij*_, only one element is non-zero, namely, $c_{ij;ij}=E\left[|Z_{ij}{|}^{2}\right]\neq 0.$ In QL, such a covariance matrix is represented by the pure state |*ij*〉 ∈ H (or the density operator ρ_*ij*_ = |*ij*〉〈*ij*|).

Now consider the compound neural network *S* = (*S*_1_, *S*_2_) with an arbitrary pattern of ROs. In QLM, its covariance matrix *C* is given by the density matrix


$$\begin{array}{l} \rho =C/TrC=\sum_{ij,km}r_{ij,km}|ij\;\rangle \langle km|. \end{array}$$

Hence, the state space of density operators acting in H is represented as the tensor product *D*(H) = *D*(H_1_) ⊗ *D*(H_2_), where H_*i*_ is the Hilbert space for the network *S*_*i*_, *i* = 1, 2.

Up to now, we have followed the standard quantum framework for a compound system *S* = (*S*_1_, *S*_2_). As usual, we can consider the marginal states of ρ generated by partial tracing,


$$\begin{array}{l} \rho_{1}=Tr_{H_{2}}\;\rho ,\rho_{2}=Tr_{H_{1}}\;\rho . \end{array}$$ (28)

In the quantum formalism, these states are interpreted as the states of the subsystems *S*_1_ and *S*_2_ of the system *S* = (*S*_1_, *S*_2_). However, in our neuronal model, we can consider the states ρ_*S*_1__ and ρ_*S*_2__ of the neuronal networks *S*_1_ and *S*_2_ in the absence of signaling between them. We remark that the network *S*_1_ ⊗ *S*_2_ is created via activation of the cross-connection between neurons in *S*_1_ and *S*_2_, and the inter-network connections contribute to signaling between *S*_1_ and *S*_2_ only indirectly. In short, the correlations $c_{S_{m};ij}=E\left[z_{m,i}{\bar{z}}_{m,j}\right],m=1,2,$ cannot be reconstructed from the covariance matrix *C* of correlations in *S*_1_ ⊗ *S*_2_, and the subsystems' QL states ρ_*S*_*m*__, *m* = 1, 2, are not equal to the marginal states ρ_*m*_.

We considered the observables *A*_1_ and *A*_2_. If only the circuit 𝔰_*ij*_ is activated, then *A*_1_ = *i, A*_2_ = *j* with probability 1. In QLM, they are represented by the operators, which are diagonal in the basis (|*ij*〉). Then we can use the construction from Sections 6 and 7—observational entanglement. In particular, we created the tensor-product structure,


$$\begin{array}{l} H=H_{1}\;⊗\;H_{2}. \end{array}$$

## 9 Ephaptic entanglement

This is an appropriate place to point out *ephaptic coupling between neuronal structures in the brain* (Schrödinger, 1935; Buzsáki et al., 2012; Anastassiou et al., 2011). This coupling enables communication between neurons that differs from direct systems based on physical connections, such as electrical synapses and chemical synapses. Through this mechanism, signals in nerve fibers can be correlated as a result of local electric fields. Ephaptic coupling can generate synchronization of action potential firing in neurons (Fröhlich and McCormick, 2010).

Recently, this coupling was highlighted in article (Radman et al., 2007) on modeling non-local representation of memories:

“*It is increasingly clear that memories are distributed across multiple brain areas. Such ‘engram complexes' are important features of memory formation and consolidation. Here, we test the hypothesis that engram complexes are formed in part by bioelectric fields that sculpt and guide neural activity and tie together the areas that participate in engram complexes. … Our results … provide evidence for in vivo ephaptic coupling in memory representations*.”

Our QL formalism describes such non-local correlations and, in particular, memories distributed across multiple brain areas. Such non-local memories are encoded in QL states. Here, we again recall our basic conjecture that the brain explores the QL representation, that is, it operates not with oscillations in neuronal networks but with the corresponding covariance matrices.

Thus, the creation of the entanglement-generating network *S*_ ⊗ _ is *a process*. At each instance in time, the character of signaling between neurons in the circuits 𝔰_1, *i*_ and 𝔰_2, *j*_ plays a crucial role in the generation of a new circuit 𝔰_*ij*_ and a specific state of *S*_⊗_, entangled or not.

## 10 Toward experimental verification of mental entanglement

### 10.1 Experimental framework for entanglement of neuronal networks

Here, we consider the simplest case of the model for entanglement of two neuronal networks presented in (see also Khrennikov et al., 2025b). In this way, we can generate only a restricted set of entangled states (in contrast to the general model). Nevertheless, some examples of entangled states can still be obtained.

The nodes of the compound network *S*_1_ ⊗ *S*_2_ are given by the pairs of nodes of the networks *S*_1_ and *S*_2_,


$$\begin{array}{l} {𝔰}_{ij}=\left({𝔰}_{1,i},{𝔰}_{2,j}\right), \end{array}$$ (29)

and ROs in these node-circuits are described as the random variables *Z*_*ij*_ = *z*_1, *i*_*z*_2, *j*_, where the random variables *z*_1, *i*_ and *z*_2, *j*_ describe ROs in node-circuits of corresponding neuronal networks. Hence, the elements of the cross-covariance matrix *C* are given as


$$\begin{array}{l} c_{ij,km}=E\left[Z_{ij}{\bar{Z}}_{km}\right]=E\left[z_{1,i}z_{2,j}{\bar{z}}_{1,k}{\bar{z}}_{2,m}\right] \end{array}$$ (30)

Consider now two spatially separated areas in the brain and two sets of electrodes 𝔰_1, *i*_, *i* = 1, …, *N*_1_, and 𝔰_2, *j*_, *j* = 1, …, *N*_2_, coupled to the respective areas. Their outputs correspond to random variables *z*_1, *i*_ and *z*_2, *j*_. Under the assumption of ergodicity, we can identify statistical averages with time averages. We center the random variables by subtracting their averages. We calculate the cross-covariance matrix. Then, by using a test of entanglement (Section 10), we determine whether the corresponding density matrix represents an entangled or separable state.

Since directly measuring correlations between signals generated by individual neurons in the brain is experimentally complicated, it is natural to employ EEG/MEG techniques to measure correlations between signals generated in spatially and functionally separated brain areas.

We also mention fMRI as a possible modality, but its limited temporal resolution makes it less suitable for detecting fast or transient correlations as required for entanglement. EEG or MEG would be more appropriate owing to their millisecond-scale resolution.

*Note for implementation*.

Practical EEG/MEG implementation details (preprocessing, spectral estimation, window length *T*, and statistical controls) are summarized in Section 10.3; see also standard references (Mitra and Pesaran, 1999; Khrennikov, 2017; Percival and Walden, 1993).

### 10.2 Supporting arguments for using EEG measurements for entanglement determination

EEG measurements consistently reveal fast, long-range synchronization of neural activity across widely separated cortical regions (Halverson, 2021). This means that brain areas located several centimeters apart—sometimes even across hemispheres—can enter into phase-locked oscillatory states. Such long-range correlations have been observed particularly in the beta (13–30 Hz) and gamma (30–100 Hz) frequency bands, which are known to underlie integrative cognitive functions such as attention, perception, and working memory. Studies using coherence and phase-synchrony analyses have demonstrated that EEG signals recorded from distant electrodes can show high phase coherence, indicating functional coupling among neuronal populations.

Therefore, it is natural to use data collected through EEG measurements to detect “mental entanglement,” and we plan to do this in our future experimental and theoretical studies; in short, classical EEG data will be analyzed with quantum information tools.

This is a suitable place to mention study (Shor et al., 2021b), where the QLM framework was applied to classical EEG data with the aim of improving the diagnosis of mental disorders. In this work, EEG signals are treated within the formalism of Bohmian mechanics, leading to the introduction of a QL potential.

This construct is inspired by the Bohmian quantum potential V, which in quantum mechanics expresses the non-local dynamics of a system. Even in physics, this potential is often regarded as a purely informational quantity, not a physical field in the ordinary sense. A similar informational interpretation can be assigned to the QL potential defined in Shor et al. (2021b).

**Figure 4:**
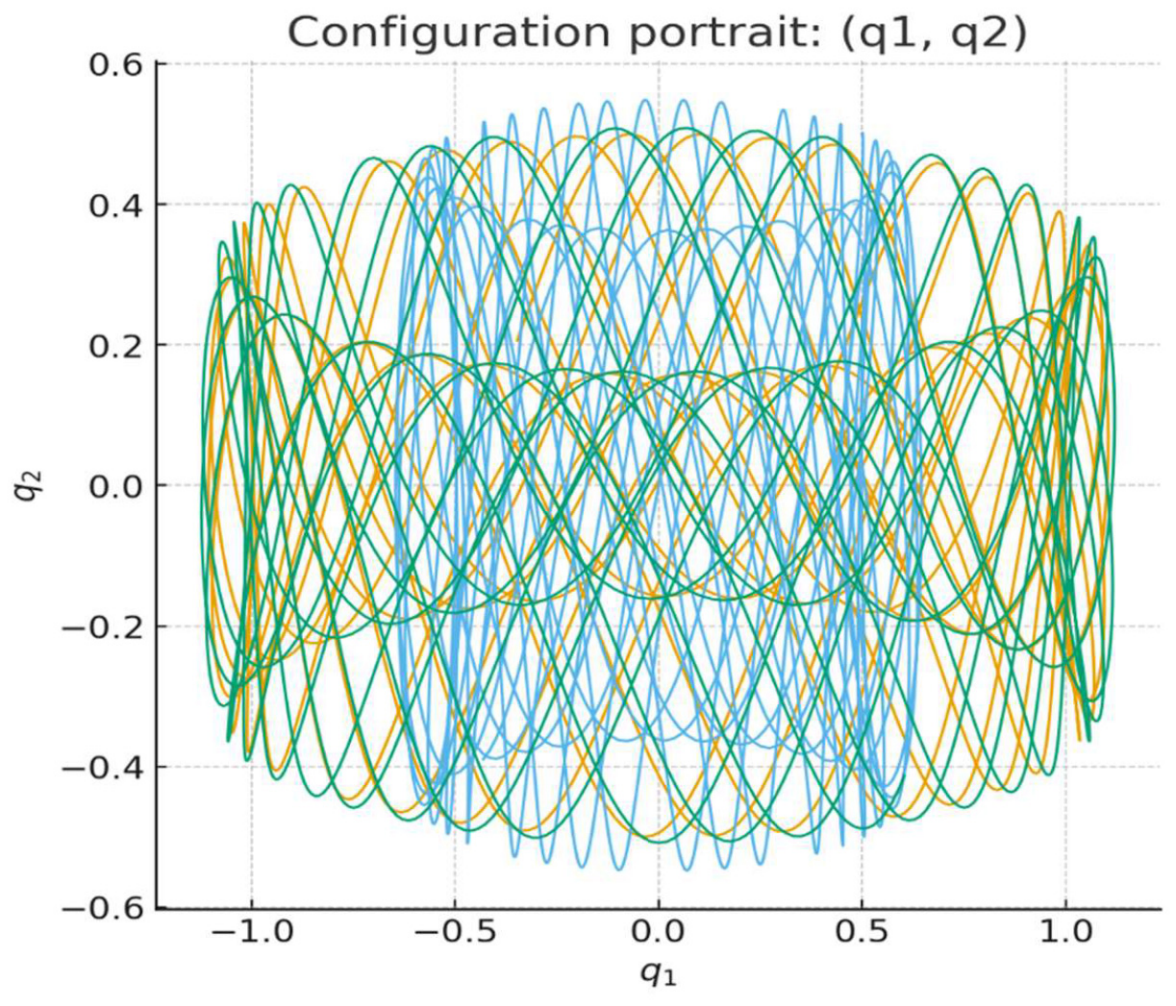
Trajectories in the configuration space of solutions of the system of coupled harmonic oscillators under consideration.

**Figure 5:**
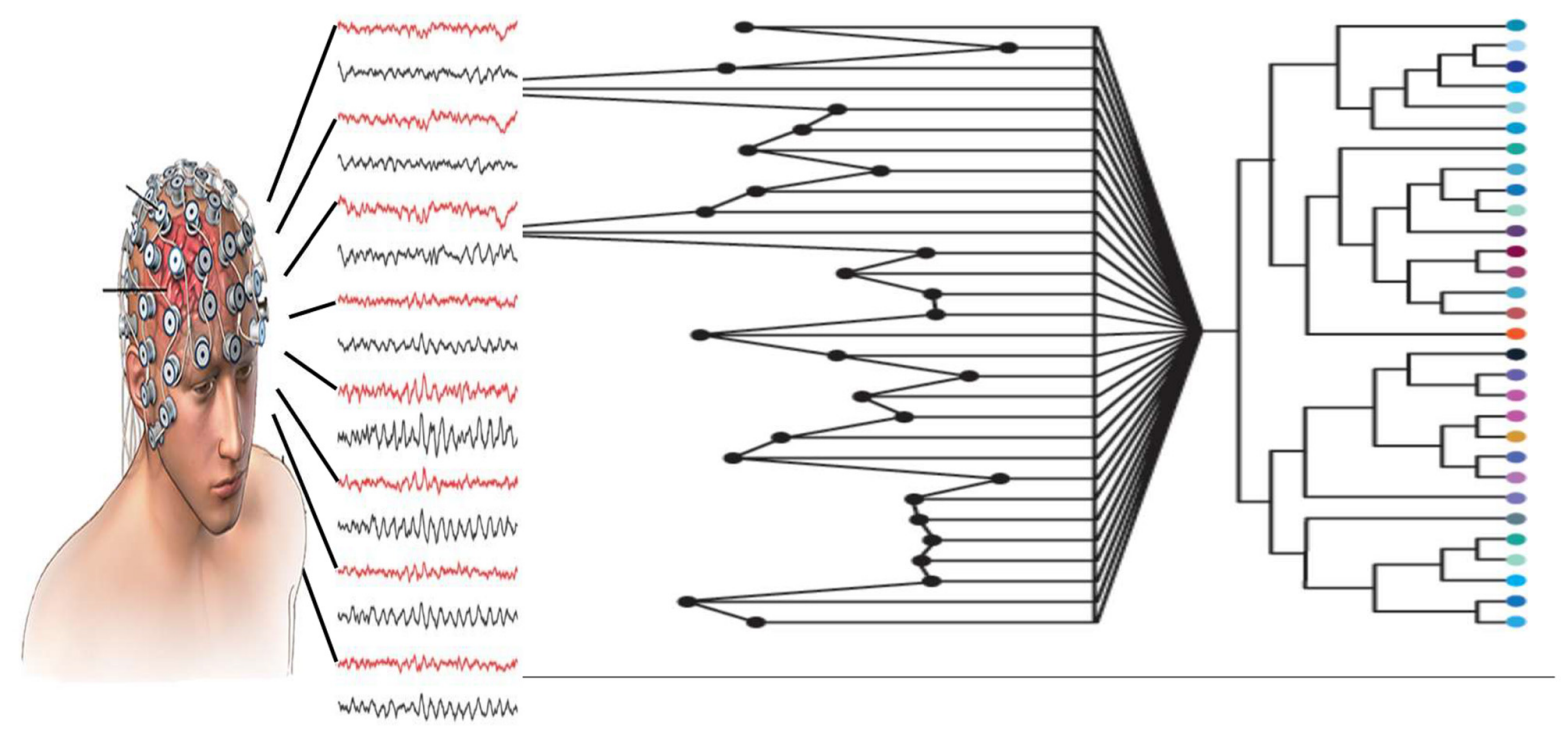
Holistic and non-local representation of EEG data generated with clustering algorithms. QL potential constructed from this dendrogram representation and reflecting entanglement mirrors how the brain processes information non-locally across dense neural networks. Dendrogramic representation transfer EEG signal into quantized output.^a^ ^a^This illustration is borrowed from the presentation of Felix Benninger at conference QIP25 (Växjö, Sweden, June 2025) with his permission.

More specifically, the QL potential in that study is not defined in physical space, but rather in a non-Archimedean (p-adic) number space. This space is generated by clustering algorithms applied to EEG data, which produce tree-like hierarchical and discrete structures analogous to neural networks, see Figure 4. In this way, the clustering and dendrogram representations of EEG signals can be seen as a form of hierarchical discretization of the brain's electrical activity (Figure 5).

Within this framework, the EEG time series are transformed into QL probability amplitudes defined on the dendrograms. From these amplitudes, the quantum-like potential is computed. This potential captures the informational and dynamical organization underlying non-local brain activity, rather than merely reflecting the electrical amplitudes recorded by EEG (Figure 5).

The QL potential may thus serve indirectly as a measure of entanglement, since Bohmian mechanics—although deterministic—encodes non-local dependencies between subsystems. However, entanglement can be more directly characterized through the violation of Bell-type inequalities (see Section 12). Our numerical simulations (Shor et al., 2021a) revealed such violations of Bell inequalities, indicating the presence of non-classical correlations. Nonetheless, equivalent analyses have not yet been performed on real EEG data, and this remains an open direction for future research.

The key result Shor et al., (2021b) is that this QL potential can accurately discriminate between EEGs of healthy individuals and those of patients diagnosed with depression, schizophrenia, and cognitive decline. In other words, it functions as a diagnostic biomarker that captures subtle, system-level differences in brain organization that are not easily detected by conventional EEG analysis. This finding was interpreted as evidence of QL structures in information processing in the brain, rather than literal quantum physical processes, may underlie patterns of brain coordination and dysfunction.

### 10.3 EEG/MEG implementation: minimum requirements

Prefer *source-space* analyses and state whether leakage correction was applied [e.g., symmetric orthogonalization (Pinotsis and Miller, 2023)]. Note that sensor-space signals are linear mixtures of underlying sources; therefore, source-space analyses with leakage correction are preferred wherever possible. Report the reference scheme, artifact handling (e.g., Independent Component Analysis (ICA) for EOG/EMG), and filtering (including notch; Mitra and Pesaran, 1999). For zero-lag confounds, accompany coherence or Phase-Locking Value (PLV) with imaginary coherence and/or wPLI [with limitations noted (Colclough et al., 2015; Nolte et al., 2004)]. These analyses quantify statistical dependence and do not by themselves establish directionality or causation. Specify spectral estimation (Welch or multitaper) and effective degrees of freedom; choose *T* to cover 5–10 cycles (Δ*f* ≈ 1/*T*; Khrennikov, 2017; Thomson, 1982; Percival and Walden, 1993). Use matched surrogates (trial shuffling or phase randomization) and correct for multiple comparisons (Mitra and Pesaran, 1999). List reproducibility items: SNR, number of tapers/segments, leakage correction, inverse model or regularization, and whether analyses were performed in sensor or source space.

### 10.4 Parallels between classical EEG-based neurophysiological approaches and QLM of cognition

It is important to emphasize that our QL modeling framework shares methodological parallels with well-established neurophysiological tools used in EEG/MEG-based studies (Vinck et al., 2011; Friston, 2011; Lachaux et al., 1999). Both approaches rely on the analysis of covariance structures and correlations among signals.

#### 10.4.1 Functional connectivity in neuroscience

Functional connectivity refers to the statistical dependencies between spatially distinct neuronal populations. It is formally defined as the *temporal correlation* between neurophysiological signals recorded at different sites in the brain, such as EEG channels.

Mathematically, common measures of functional connectivity include:

*Covariance*.

For two time series *x*(*t*) and *y*(*t*), the covariance is defined as:


$$\begin{array}{l} Cov\left(x,y\right)=\frac{1}{T}\overset{T}{\underset{t=1}{\sum }}\left(x\left(t\right)-\mu_{x}\right)\left(y\left(t\right)-\mu_{y}\right), \end{array}$$ (31)

where μ_*x*_, μ_*y*_ are the mean values of *x*(*t*) and *y*(*t*), and *T* is the total number of time points.

*Pearson correlation coefficient*.


$$\begin{array}{l} r_{xy}=\frac{Cov\left(x,y\right)}{\sigma_{x}\sigma_{y}} \end{array}$$

where σ_*x*_, σ_*y*_ are the standard deviations of the signals.

*Coherence*.

Coherence measures frequency-specific linear correlations between signals:


$$\begin{array}{l} C_{xy}\left(f\right)=\frac{|S_{xy}\left(f\right){|}^{2}}{S_{xx}\left(f\right)S_{yy}\left(f\right)} \end{array}$$

where S_*xy*_(*f*) is the cross-spectral density, and S_*xx*_(*f*) and S_*yy*_(*f*) are the auto-spectral densities. (*Estimator notes:* Welch or multitaper estimators are commonly used; see Khrennikov, 2017; Thomson, 1982; Percival and Walden, 1993; Srinivasan et al., 2007).

*Phase-locking value (PLV)*.

PLV quantifies phase synchronization between two signals:


$$\begin{array}{l} PLV=|\frac{1}{T}\overset{T}{\underset{t=1}{\sum }}e^{i\left(\phi_{x}\left(t\right)-\phi_{y}\left(t\right)\right)}| \end{array}$$

where ϕ_*x*_(*t*) and ϕ_*y*_(*t*) are the instantaneous phases, typically extracted using the Hilbert transform or wavelet methods [following (Friston, 2011)].

*Scope of functional connectivity (FC) metrics*.

These metrics quantify statistical dependence but not directionality or causation; directional inferences require separate analyses and explicit assumptions.

*Zero-lag confounds and robust metrics*.

To mitigate volume conduction and common reference effects, the imaginary part of coherency and/or the weighted phase-lag index (wPLI) should be reported alongside classical metrics (Colclough et al., 2015; Nolte et al., 2004). However, these approaches do not eliminate all leakage in sensor space; whenever possible, analyses should be performed in source space with leakage correction.

*Applications*.

FC networks are generated by computing these measures pairwise across brain regions, producing a connectivity matrix that is subsequently analyzed for modularity, hub architecture, and network dynamics. These methods are extensively applied to investigate cognition, neurological disorders, and stimulus-driven responses (Bruns, 2004; Vinck et al., 2011; Bastos and Schoffelen, 2016; Mitra and Pesaran, 1999).

#### 10.4.2 Parallels with QLM

In the QL framework, similar mathematical constructs arise naturally. The density matrix or generalized covariance matrix represents probabilistic dependencies among cognitive observables, analogous to FC matrices in EEG studies. Furthermore, off-diagonal terms in QL states encode interference-like effects, comparable to EEG phase synchrony measures, such as PLV and coherence.

Thus, the QL formalism extends conventional measures by providing a richer probabilistic interpretation grounded in generalized state representations.

Finally, the concept of entanglement in QL modeling can be compared—at a conceptual level—with strong inter-regional correlations identified in FC analyses, where clusters of brain regions operate as integrated modules. This analogy is heuristic and does not indicate equivalence of constructs.

This is an appropriate place to note that classical signal analysis of brain function frequently employs the analytic signal representation via the Hilbert transform (see, e.g., Srinivasan et al., 2007; Mitra and Pesaran, 1999; Friston, 2011). The established use of such complex-valued representations suggests another avenue for QL-style modeling of brain dynamics. We plan to develop such a model in a future article.

#### 10.4.3 Summary of parallels

**Key takeaway**. The correspondence between EEG/MEG FC and QL constructs is *conceptual*: both capture dependencies through second-order structure (covariances/coherences vs. density matrix off-diagonals) (see Table 1). This analogy is heuristic and does *not* imply equivalence of constructs, measurement units, or causal mechanisms.

**Table 1:** Comparison of EEG/MEG-based methods and QL modeling of cognition (conceptual mapping; not a one-to-one equivalence).

**Concept**	**EEG/MEG neurophysiology**	**QL modeling of cognition**
Covariance	Covariance/correlation	Density matrix (covariances)
Synchrony	Coherence/phase-locking (PLV)	Interference (off-diagonals)
Network correlations	Functional networks	Entanglement

**Practical implications**.

- Treat FC metrics as measures of *statistical dependence*, not directionality; use source space with leakage correction and report imaginary coherency/wPLI when applicable.
- When robust FC modules persist under stringent controls, QL analyses can quantify non-separability via mixed-state entanglement measures (Section 11).

### 10.5 *In vitro* neuronal networks

*In vitro* neuronal networks are cultures of neurons that replicate key aspects of network structure and function. In such preparations, signals from individual neurons or defined circuits can be recorded directly; connectivity can be patterned and currents across connections between spatially separated subnetworks can be measured. Although experimentally demanding, these paradigms are feasible and align closely with our framework (cf. Section 10.1).

The experimental testing of QL entanglement *in vitro* is increasingly practical owing to advances in multi-electrode arrays (MEAs), which allow simultaneous stimulation and recording from dozens to hundreds of sites.

One promising approach is *patterned electrical stimulation* to impose structured correlations between distinct subpopulations—for example, time-locked or phase-modulated sequences delivered to spatially separated regions to create controlled coupling patterns.

Additionally, *pharmacological modulation* offers a complementary route, for example:

- **Bicuculline** (GABA_*A*_ antagonist) to increase network excitability and enhance synchrony;
- **Carbachol** or other acetylcholine agonists to regulate oscillatory dynamics and increase coherence.

These manipulations can serve to test QL non-separability by:

- engineering structured couplings via stimulation or pharmacology,
- recording the resulting activity with MEAs, and
- analyzing correlations using QL-inspired criteria (e.g., separability bounds).

Such protocols provide a concrete route toward evaluating QL entanglement while maintaining continuity with established neurophysiological methods. Finally, experimental confirmation of QL non-separability would support quantum-inspired computation with neuronal networks—QL neuromorphic computing (see Igamberdiev, 2004; Amati and Scholes, 2025; Igamberdiev and Brenner, 2021; Khrennikov et al., 2025b,a).

## 11 Basic quantitative measures of entanglement for mixed states

In quantum information theory, the entanglement of mixed states is quantified using various measures defined through density operators. These measures are critical for characterizing quantum correlations in composite systems described by mixed states. In the following, we summarize mixed-state entanglement measures most relevant for empirical analyses of QL states reconstructed from neural signals. In the context of QLM of cognition, such measures may be applied to examine “mental entanglement” and complex cognitive interdependencies.

### 11.1 Terminology

Throughout this section, “entanglement” refers to the non-separability of the *QL* state ρ, constructed from classical neural signals (e.g., EEG/MEG/fMRI-derived time series). We do not suggest microscopic quantum entanglement in brain tissue; outcomes depend on the selected subsystem partition and the measurement basis used to define ρ and the partial transpose.

We start with the definition of the *von Neumann entropy* of a generally mixed state given by a density matrix:


$$\begin{array}{l} S\left(\rho \right)=-Tr\left(\rho \log\rho \right) \end{array}$$

It measures the degree of mixedness of the state; *S*(ρ) = 0 if and only if ρ is a pure state. Hence, it can be used as a measure of the purity of a quantum (or QL) state.

Now let ρ be the density operator of a bipartite system on Hilbert space H_*A*_ ⊗ H_*B*_.

### 11.2 Entanglement entropy

For a pure bipartite state ρ_*AB*_= |ψ_*AB*_〉〈ψ_*AB*_|, the entanglement entropy is defined as the von Neumann entropy of the reduced state:


$$\begin{array}{l} S_{A}=S\left(\rho_{A}\right),\;\rho_{A}=Tr_{B}\left(\rho_{AB}\right) \end{array}$$ (32)

This quantity measures the degree of entanglement between subsystems *A* and *B* for pure bipartite states. For pure global states, entanglement is determined by the entropy of the reduced state: *S*(ρ_*A*_) > 0 if and only if ρ_*AB*_ is entangled [and *S*(ρ_*A*_) = 0 if it is a product state]. In contrast, *S*(ρ) = 0 or the linear entropy $S_{L}\left(\rho \right)=1-Tr\rho^{2}=0$ only confirms that the global state is pure, not whether it is entangled across a given bipartition.

In cognitive and neuronal data, however, pure QL states are not expected: noise, non-stationarity, and averaging typically yield mixed states. Therefore, mixed-state entanglement measures are required.

### 11.3 Negativity and logarithmic negativity

Negativity quantifies entanglement by examining the partial transpose of the density matrix:


$$\begin{array}{l} N\left(\rho \right)=\left(∥\rho^{T_{B}}{∥}_{1}--\;1\right)/2 \end{array}$$

where $\rho^{T_{B}}$ is the partial transpose with respect to the subsystem *B* and ||$\rho^{T_{B}}{∥}_{1}$ is the trace norm of $\rho^{T_{B}}$.

Logarithmic negativity is defined as:


$$\begin{array}{l} E_{N}\left(\rho \right)=\log_{2}\;∥\rho^{T_{B}}{∥}_{1} \end{array}$$

These are standard entanglement monotones for mixed states; the partial transpose is taken with respect to the chosen subsystem in the product basis.

### 11.4 Concurrence (two-qubit systems)

For two-qubit mixed states ρ, concurrence is defined as:


$$\begin{array}{l} C\left(\rho \right)=max\left(0,\lambda_{1}-\lambda_{2}-\lambda_{3}-\lambda_{4}\right) \end{array}$$

where λ_*i*_ are the square roots of the eigenvalues (in decreasing order) of:


$$\begin{array}{l} R=\rho \left(\sigma_{y}⊗\sigma_{y}\right)\rho^{*}\left(\sigma_{y}⊗\sigma_{y}\right)\; \end{array}$$

with ρ^*^ denoting complex conjugation and σ_*y*_, the Pauli y matrix.

These measures of entanglement quantify quantum correlations between systems. In quantum theory, there is also a measure used to capture combined classical–quantum correlations.

For two-qubit systems, concurrence corresponds to the entanglement of formation.

### 11.5 Quantum mutual information

For mixed states, quantum mutual information measures total correlations:


$$\begin{array}{l} I\left(A:B\right)=S\left(\rho_{A}\right)+S\left(\rho_{B}\right)-S\left(\rho_{AB}\right) \end{array}$$

If *I*(*A*:*B*) = 0, the two systems are uncorrelated (neither classical nor QL correlations). If *I*(*A*:*B*) > 0, there are correlations, but this measure does not distinguish QL from classical components and is not an entanglement monotone.

However, mutual information is not a measure of entanglement, because

- **Non-zero for Separable States:** Even separable (non-entangled) mixed states can yield non-zero mutual information because of classical correlations.
- **Entanglement Monotonicity:** Valid entanglement measures must not increase under local operations and classical communication. Mutual information fails this criterion because it quantifies all correlations, not exclusively quantum ones.

In this section, we discuss deep foundational questions of quantum mechanics—specifically, the various interpretations of the relationship between *quantum* and *classical entanglement* and how these discussions are projected within *quantum-like (QL)* approaches to cognition and social systems. Readers who are not primarily interested in the foundations of quantum theory may omit this section. Nevertheless, even for such readers, it may be useful to gain some sense of the current debates in quantum foundations and the diversity of their interpretations.

## 12 Quantum, classical, and mental entanglement, correlations, and Bell inequality

Formally, entanglement is defined as *state non-separability* [see Section 8, violation of equality (Equation 26)]. This theoretical notion plays a central role in quantum information theory, particularly in quantum computing. However, providing a physically meaningful interpretation of this abstract definition remains a complex foundational problem. One of the central issues is clarifying the relationship between *quantum* and *classical* entanglement (Bruza et al., 2015; Spreeuw, 1998, 2001). The latter refers to correlations between different degrees of freedom of the *same classical field*. For example, polarization and spatial modes of an optical beam can be correlated. Consider a laser beam where polarization depends on the transverse spatial mode:


$$\begin{array}{l} E\left(x,y\right)=f\left(x,y\right)H+g\left(x,y\right)V, \end{array}$$

which cannot be factorized into a “pure polarization” and a “pure spatial mode.”

Now consider a pair of beams with equal intensity and orthogonal polarization:


$$\begin{array}{l} |\Psi )=\frac{1}{\sqrt{2}}\left(|x_{1}V)+|x_{2}H)\right) \end{array}$$ (33)

and note, we consider this pair of beams, *x*_1_ and *x*_2_, *as a single object*. As a single entity, this beam pair is neither in a single, pure polarization state, nor in a single, pure position state.

Thus, *classical entanglement* is the *non-separability of certain degrees of freedom* in the classical electromagnetic field (or other classical systems).

A useful review of the quantum–classical entanglement problem can be found in Spreeuw (2001), which distinguishes three types of physical entanglement:

- **Non-local quantum entanglement**—entanglement between distinct physical entities (particles, optical beams, atomic ensembles, etc.). Example: two two-level systems in a Bell state.
- **Intra-mode (local) quantum entanglement**—entanglement between different properties of a *single* entity, such as different degrees of freedom within one atom.
- **Intra-mode classical entanglement**—entanglement between distinct properties of a single optical beam.

One might say that non-local entanglement concerns states on which two *independent measurements* can be performed, each producing its own statistical distribution, but showing strong correlations when compared. Local entanglement, by contrast, refers to states where such independent measurements are impossible. Initially, this locality distinction was seen as a defining feature of *classical entanglement* (Bruza et al., 2015; Spreeuw, 1998). However, local *quantum* entanglement is also “local” in this sense.

This raises a fundamental question: *What truly distinguishes genuine quantum entanglement from classical entanglement?*

Different authors and research groups have proposed diverse answers. The most widely cited position is that of Spreeuw in his influential article in his influential article Bruza et al., (2015), often invoked by experimentalists as a foundational justification for optical analogs of quantum phenomena:

“It is found that the model system (the classical electromagnetic field) can successfully simulate most features of entanglement but fails to simulate quantum non-locality. Investigations of how far the classical simulation can be pushed show that quantum non-locality is the essential ingredient of a quantum computer, even more so than entanglement. The well-known problem of exponential resources required for a classical simulation of a quantum computer is also linked to the non-local nature of entanglement, rather than to the non-factorizability of the state vector.”

The last sentence reflects on the broad range of *quantum-inspired* applications of classical optical entanglement in information theory and computation (Spreeuw, 2001). Spreeuw further writes (Bruza et al., 2015):

“However, the (classical–quantum) analogy fails to produce effects of quantum non-locality, thus signaling a profound difference between two types of entanglement: (i) ‘true' multiparticle entanglement and (ii) a weaker form of entanglement between different degrees of freedom of a single particle. Although these two types look deceptively similar in many respects, only type (i) can yield non-local correlations. Only type (ii) entanglement has a classical analogy.”

The reference to *quantum non-locality*—or “spooky action at a distance”—is widespread in the quantum community. It is often cited to emphasize the essential difference between quantum and classical physics and to highlight the “mystery” of quantum theory, encapsulated in Feynman's famous statement (Korolkova and Leuchs, 2019):

“I think I can safely say that nobody understands quantum mechanics.”

However, invoking “spooky action at a distance” is not acceptable within the QLM framework. Such an interpretation would blur the boundary between cognitive psychology and parapsychology or studies of anomalous phenomena. Although such topics may have their own value, they lie outside the scope of QLM. Therefore, within QLM, we seek *non-mysterious* features of “quantumness” that do not rely on action at a distance.

One of the authors of this article has proposed an alternative approach to distinguishing quantum from classical entanglement (Feynman, 1967) based on Bohr's notion of a phenomenon (Khrennikov, 2020; Bohr, 1987; Plotnitsky, 2009):

“…in actual experiments, all observations are expressed by unambiguous statements referring, for instance, to the registration of the point at which an electron arrives at a photographic plate. … the appropriate physical interpretation of the symbolic quantum mechanical formalism amounts only to predictions, of determinate or statistical character, pertaining to individual phenomena…” (Bohr, 1987, v.2, p. 64)

As noted in Feynman (1967):

“Although quantum theory provides statistical predictions, its observables generate *individual phenomena*. The discreteness of detection events is the fundamental feature of quantum physics, justifying the existence of quantum systems as carriers of quanta.”

This distinction is supported by Grangier's experiment on “the existence of photons” (Plotnitsky, 2012), which established a clear operational boundary between quantum and semiclassical regimes. The experiment demonstrated that detector clicks correspond—via a cascade amplification process—to discrete quantum entities, *photons*.

By contrast, in *classical optics*, measurements are continuous, corresponding to signal *intensities* rather than discrete detection events. Hence, state non-separability plus discreteness of measurement outcomes can be seen as the true operational markers of “quantumness.” This view aligns well with the principles of QLM of cognition.

In applications of QL cognition, it is convenient to follow the position of Eberly et al. (2016), who argue that associating entanglement exclusively with quantum theory is unnecessary. According to them:

“Entanglement is a vector-space property, present in any theory with a vector-space framework.”

They reject the need to distinguish between “quantum” and “classical” entanglement, defining it simply as *non-separability of sums of product states across distinct vector spaces*.

Our notion of mental entanglement fits naturally within this vector-space perspective. In EEG experiments, we aim to detect precisely such *mental state non-separability*. Since EEG signals are classical electrical signals, these experiments would demonstrate *classical entanglement*; detection is based on *signal intensities*, not on discrete events as in photon counting.

This raises a further question: Can we design neurocognitive experiments involving joint measurements of observables with *discrete outcomes*? Such experiments could provide evidence for *mental entanglement* more closely analogous to *quantum entanglement* than that detected through EEG intensity correlations. One way is the discretization of EEG signals based on clustering algorithms (see Section 10.2).

Finally, we emphasize that the relationship between quantum and classical entanglement remains an open question in modern physics (Spreeuw, 2001):

“The nature of the phenomenon once coined ‘classical entanglement' is still widely debated. While the vector-space framework appears consistent for all states considered classically entangled, its implications differ across physical systems. Schrödinger introduced quantum mechanics as wave mechanics; the language of wave optics naturally incorporates features such as coherence, interference, and superposition—properties often associated with quantum systems. Nevertheless, the main open and controversial question remains: which, if any, of these features are *genuinely quantum*?”

Therefore, the interrelations among quantum, classical, and mental entanglement remain subjects for further debate. These discussions can be viewed as the first steps toward developing a new discipline: the foundations of quantum-like cognition and decision theory.

### 12.1 Bell inequalities and “superstrong” correlations

A key quantitative measure of entanglement is the *degree of violation* of Bell inequalities. For some quantum states, the pairwise correlations of jointly measurable observables violate inequalities that classical observables must satisfy. In physics, such phenomena are described as “*superstrong quantum correlations*.”

A quantum state capable of violating Bell inequalities must be *entangled*, but the relationship is subtle. In his celebrated article, Werner (1989) constructed *mixed entangled states* that *do not* violate Bell inequalities—showing that entanglement does not always imply Bell violation.

This relationship can be summarized as follows:

- Entanglement ⇒ may violate Bell inequalities (but not necessarily)
- Non-entangled ⇒ never violates Bell inequalities

In quantum physics, such quantum correlations are often attributed to *non-locality*—Einstein's “spooky action at a distance.” An alternative interpretation traces them to the *contextuality* of quantum observables. This idea, originally explored by Bell, lacks a clear physical explanation for the origin of contextuality, leading Bell himself to favor the non-local interpretation.

Within QLM, however, *contextuality* provides a more natural framework than non-locality. Bell inequalities are understood as *non-contextual inequalities*, and their violation is interpreted as a *signature of contextuality*.

In cognitive psychology and decision-making research, Bell-type inequalities have also been explored, beginning with early foundational and experimental studies (Conte et al., 2008) [see for later experiments (Asano et al., 2015; Dzhafarov and Kujala, 2012; Cervantes and Dzhafarov, 2018; Basieva et al., 2019; Gallus et al., 2022, 2023; Khrennikova, 2025)]. As in physics, these investigations are complex both conceptually and empirically.

## 13 Conceptual differences and added value of the QL framework

While there are important methodological parallels between our QL framework and classical neurophysiological approaches, it is essential to emphasize the fundamental conceptual innovations that distinguish the QL model.

Most classical models in neuroscience—such as *Principal Component Analysis* (PCA), *ICA*, and *Integrated Information Theory* (IIT)—are based on analyzing statistical dependencies or decomposing neural signals into independent or maximally informative components. These approaches often assume linearity, Gaussianity, or specific information–theoretic structures, and they generally function within the framework of classical probability theory.

In contrast, the QL framework introduces the formalism of *operator-based observables* and tensor-product *structures*, adapted from quantum theory but applied to macroscopic neuronal information processing. These mathematical tools allow the formal representation of several fundamental cognitive phenomena, including:

- **Contextuality:** The outcome of cognitive measurements depends on the context, similar to the contextuality of quantum measurements.
- **Incompatibility:** Certain cognitive observables cannot be simultaneously measured or precisely assigned, reflecting uncertainty and complementarity in cognition.
- **Entanglement:** Complex dependencies and holistic cognitive states can be modeled through entangled QL states.

Mathematically, these phenomena are naturally expressed using non-commuting operators and composite Hilbert spaces:


$$\begin{array}{l} H_{total}=H_{1}⊗H_{2}\; \end{array}$$

where the subsystems correspond to distinct cognitive or neural components.

The density operator (or generalized state) in the QL model encodes both classical correlations and quantum-like correlations (entanglement), extending beyond covariance-based approaches such as PCA and ICA.

*Added value*

By enabling such generalized probabilistic structures, the QL approach formally extends classical theories. It provides novel ways to model:

- Non-classical interference effects in cognition.
- Strong contextual dependencies in decision-making.
- Holistic, system-level processes are inaccessible to classical decompositions.

This conceptual generalization bridges neurophysiological signal processing with higher-level cognitive modeling, offering an integrated framework for studying cognition beyond traditional statistical tools.

## 14 Concluding remarks

This article represents a step toward constructing a conceptual and mathematical bridge between oscillatory cognition—defined as the rhythmic activity of neuronal networks—and QL models of cognition, which have been successfully applied to explain contextuality, interference, and entanglement-like effects in human behavior and decision-making. This bridge relies on a fundamental correspondence: QL mental states are represented by density operators and QL observables by quadratic forms, both of which are mathematically grounded in the covariance structure of ROs in neural circuits. These constructions are developed within the framework of PCSFT, which extends beyond the standard quantum formalism (Khrennikov, 2005, 2006a, 2014a, 2024a).

Previous work (Khrennikov et al., 2025a) has suggested that PCSFT provides a viable interpretational and computational foundation for QL representations of cognitive phenomena. Here, we focus on one of the most conceptually challenging issues: *the formalization of mental entanglement—a cognitive analog of quantum entanglement*—which we consider crucial for modeling integrative mental processes involving distributed and interacting brain networks.

In quantum information theory, entanglement is central to the non-classical correlations underlying quantum computational advantage. While the physical meaning of entanglement remains debated—particularly regarding separability and locality—there has been increasing interest in an alternative formulation: *observational entanglement*. This approach avoids problematic metaphysical assumptions and emphasizes statistical correlations observed through measurements. We adopt this perspective as a more transparent entry point into modeling cognitive entanglement.

We then proceed to explore state entanglement in the QL framework, treating entangled QL states as representations of the joint activity of spatially and functionally separated neuronal assemblies. In this context, *mental entanglement* provides a natural mechanism for feature binding—the brain's capacity to integrate disparate perceptual or cognitive contents (e.g., color, shape, and motion) into unified conscious experiences. This formulation suggests a candidate QL solution to the binding problem, long regarded as one of the central unsolved questions in neuroscience and cognitive science.

Moreover, the introduction of mental entanglement offers a speculative yet potentially fruitful path toward addressing aspects of the *hard problem of consciousness*—specifically, how and why certain brain processes give rise to subjective experience. While our approach does not resolve the hard problem, it aligns with perspectives proposing that consciousness may involve non-classical, globally coherent states that resist decomposition into strictly local mechanisms. If mental entanglement reflects a form of non-local coherence in brain function, it may point to a formal structure compatible with integrated information and global workspace theories, enriched by QL formalism.

In cognitive neuroscience, numerous studies have shown that neuronal oscillations—particularly in the gamma, theta, and beta bands—are associated with integrative functions such as memory binding, attentional selection, and conscious access. Our model establishes a bridge between these empirical findings and QL cognitive models by interpreting such oscillatory patterns as classical fields whose covariance structures define the QL states and observables. This offers a testable link between neurophysiological processes and the abstract mathematical structures of QL cognition.

We further hypothesize that entangled QL states derived from ROs may underlie enhanced computational capacities in specific neural subsystems, particularly within the cerebral cortex and hippocampus. This aligns with evidence of high-performance integrative processing in these regions and indicates a deeper role for QL representations in modeling cognitive efficiency.

In Section 10.3, we outline preliminary experimental designs aimed at indirectly detecting signatures of mental entanglement using EEG/MEG methodologies, focusing on correlation structures in neural signals that diverge from classical expectations. Although speculative, these tests are intended to guide future empirical investigations.

We emphasize once again that QL structure is a statistical organization of macroscopic neural activity in a Hilbert-space description rather than microscopic quantum coherence. The relevant signals are ensemble-level covariances generated by classical coupling within and between cortical areas. Such structures are known to *persist under noise because they concern collective variables, not single-unit precision*. Our QL indices are scale-robust—uniform broadband power changes largely affect overall scale without altering non-separable structure—and they vary continuously with the underlying statistics, so realistic noise reduces magnitude rather than producing spurious effects. As an internal negative control, when the observables commute, the indices are expected to approach zero; when they do not, order effects and non-separability remain testable despite noise. Replicating the same pattern across EEG and MEG further supports the view that these phenomena track macroscopic organization rather than fragile microscopic states. Thus, in our framework, “survival under noise” follows from QL phenomena being emergent, population-level regularities grounded in classical interactions.”

In conclusion, the development of the mental entanglement formalism reinforces the broader conjecture that the brain employs QL representations in cognitive processing. This framework opens the door to a deeper interdisciplinary synthesis—integrating neurophysiological data, quantum-inspired mathematical tools, and enduring philosophical questions in consciousness research.

## Data Availability

The original contributions presented in the study are included in the article/supplementary material, further inquiries can be directed to the corresponding author.

## Footnotes

1 See De Barros and Suppes (2009), De Barros (2012), Takahashi and Cheon (2012), Busemeyer et al. (2017), Scholes (2024), and Amati and Scholes (2025) for other models aimed at coupling neuronal and QL information processing in the brain. We especially highlight the article (Khrennikov et al., 2025b) in that we employ *generalized probability* (operational measurement) theory. This is a more general formalism than quantum theory in a complex Hilbert space. But all authors exploring QLM work within the standard quantum formalism based on complex Hilbert space. So, it is useful to justify the use of this formalism from the neurophysiological viewpoint.

2 In the real case, a Gaussian distribution is uniquely determined by its covariance matrix and mean value. But in the complex case, only a *circularly symmetric* Gaussian distribution is uniquely determined by its (complex) covariance matrix. The assumption that ROs in neuronal circuits are circularly symmetric Gaussian makes the correspondence ROs → covariance matrix one-to-one. However, there are no bio-physical arguments supporting such an assumption.

3 Although subsystem *S*_*i*_ cannot be identified with a physical network of node-circuits, for external observer (who cannot “open the brain” and see the individual neuronal structure of *S*), subsystems *S*_*i*_, *i* = 1, 2, “exist” and their existence is determined via local observations.
